# Whole Grain Products, Fish and Bilberries Alter Glucose and Lipid Metabolism in a Randomized, Controlled Trial: The Sysdimet Study

**DOI:** 10.1371/journal.pone.0022646

**Published:** 2011-08-25

**Authors:** Maria Lankinen, Ursula Schwab, Marjukka Kolehmainen, Jussi Paananen, Kaisa Poutanen, Hannu Mykkänen, Tuulikki Seppänen-Laakso, Helena Gylling, Matti Uusitupa, Matej Orešič

**Affiliations:** 1 VTT Technical Research Centre of Finland, Kuopio, Finland; 2 Department of Clinical Nutrition, Institute of Public Health and Clinical Nutrition, University of Eastern Finland, Kuopio, Finland; 3 Institute of Clinical Medicine, Internal Medicine, Kuopio University Hospital, Kuopio, Finland; 4 Department of Clinical Nutrition, Food and Health Research Centre, Institute of Public Health and Clinical Nutrition, University of Eastern Finland, Kuopio, Finland; 5 VTT Technical Research Centre of Finland, Espoo, Finland; 6 Research Unit, Kuopio University Hospital, Kuopio, Finland; University of Las Palmas de Gran Canaria, Spain

## Abstract

**Background:**

Due to the growing prevalence of type 2 diabetes, new dietary solutions are needed to help improve glucose and lipid metabolism in persons at high risk of developing the disease. Herein we investigated the effects of low-insulin-response grain products, fatty fish, and berries on glucose metabolism and plasma lipidomic profiles in persons with impaired glucose metabolism.

**Methodology/Principal Findings:**

Altogether 106 men and women with impaired glucose metabolism and with at least two other features of the metabolic syndrome were included in a 12-week parallel dietary intervention. The participants were randomized into three diet intervention groups: (1) whole grain and low postprandial insulin response grain products, fatty fish three times a week, and bilberries three portions per day (HealthyDiet group), (2) Whole grain enriched diet (WGED) group, which includes principally the same grain products as group (1), but with no change in fish or berry consumption, and (3) refined wheat breads (Control). Oral glucose tolerance, plasma fatty acids and lipidomic profiles were measured before and after the intervention. Self-reported compliance with the diets was good and the body weight remained constant. Within the HealthyDiet group two hour glucose concentration and area-under-the-curve for glucose decreased and plasma proportion of (n-3) long-chain PUFAs increased (False Discovery Rate p-values <0.05). Increases in eicosapentaenoic acid and docosahexaenoic acid associated curvilinearly with the improved insulin secretion and glucose disposal. Among the 364 characterized lipids, 25 changed significantly in the HealthyDiet group, including multiple triglycerides incorporating the long chain (n-3) PUFA.

**Conclusions/Significance:**

The results suggest that the diet rich in whole grain and low insulin response grain products, bilberries, and fatty fish improve glucose metabolism and alter the lipidomic profile. Therefore, such a diet may have a beneficial effect in the efforts to prevent type 2 diabetes in high risk persons.

**Trial Registration:**

ClinicalTrials.gov NCT00573781

## Introduction

The beneficial health effects related to consumption of whole grain [1]–[3], fish or fish oil supplements [4], [5] and polyphenol rich foods such as berries [6] are well documented. However, the synergistic effects of these foods on lipid and glucose metabolism in persons at risk for type 2 diabetes have not yet been investigated.

In epidemiological studies the intake of whole grain has been associated with lower risk of obesity, insulin resistance, elevated fasting glucose and the incidence of diabetes [7]. Rye bread induces post-prandially lower insulin response than wheat independently of the fiber content [8]–[10]. Additionally, a twelve-week consumption of low insulin response diet (rye bread and pasta) has been shown to enhance early insulin secretion in persons with metabolic syndrome [2]. This diet also modulated gene expression profile of abdominal subcutaneous tissue by down-regulating genes involved in insulin signaling and apoptosis [11]. However, while the rye bread and pasta diet did not alter the lipidomic profile of plasma, the high insulin response diet (oat-wheat bread and potato) led to increased concentrations of proinflammatory lysophosphatidylcholines (LPCs) [12]. This suggests that even a moderate dietary carbohydrate modification may affect the lipid metabolism.

Bilberries are particularly abundant in polyphenols, especially anthocyanins [13]. Growing evidence from animal studies suggests that polyphenols as well as foods and beverages rich in polyphenols may positively influence carbohydrate metabolism by attenuating postprandial glycemic responses and fasting hyperglycemia as well as by improving acute insulin secretion and insulin sensitivity [6]. Human intervention studies using berries or anthocyanin extracts have also demonstrated significant improvements in low density lipoprotein (LDL) oxidation, lipid peroxidation, total plasma antioxidant capacity and dyslipidemia [14].

Epidemiological studies have demonstrated associations between the (n-3) long-chain polyunsaturated fatty acids (PUFA), found mainly in fish, and lower prevalence of insulin resistance and type 2 diabetes [15]. However, clinical trials on (n-3) PUFA-enriched diets have so far led to conflicting results [15]–[18]. The mechanisms behind the potential beneficial effect of (n-3) PUFA on glucose metabolism are poorly understood. The evidence so far points to the role of insulin receptor signaling [19], inflammation [20], [21], cell membrane fatty acid composition [22], [23], circulating hormones and adipocytokines [18] or G protein-coupled receptor 120 [24]. By applying a lipidomics approach we have recently shown that an eight-week consumption of fatty fish four to five times per week led to decreased plasma concentrations of potential mediators of lipid-induced insulin resistance and inflammation, including ceramides, diacylglycerols and LPCs [25]. Lipids are known to play a central role in the progression of glucose metabolism towards diabetes [26]. The emergence of lipidomics has enabled the global study of lipids in cells, tissues and biofluids, and revitalized the study of lipids in the context of nutrition research and clinical biomarker discovery [27].

Herein we investigate the effects of whole grain and low insulin response grain products, fatty fish, and bilberries on glucose metabolism and plasma lipidomic profile in individuals with the impaired fasting glucose (IFG) or impaired glucose tolerance (IGT) and features of metabolic syndrome. We also aimed to study whether the increase in plasma eicosapentaenoic acid (EPA) and docosahexaenoic acid (DHA) content is related to the improved glucose metabolism.

## Materials and Methods

The protocol for this trial and supporting CONSOT checklist are available as supporting information; see Checklist S1 and Protocol S1.

### Participants and study design

Participants volunteered to the study and gave their written informed consent. The study plan was approved by the Research Ethics Committee, Hospital District of Northern Savo. The intervention was performed in accordance of Helsinki Declaration.

**Figure 1:**
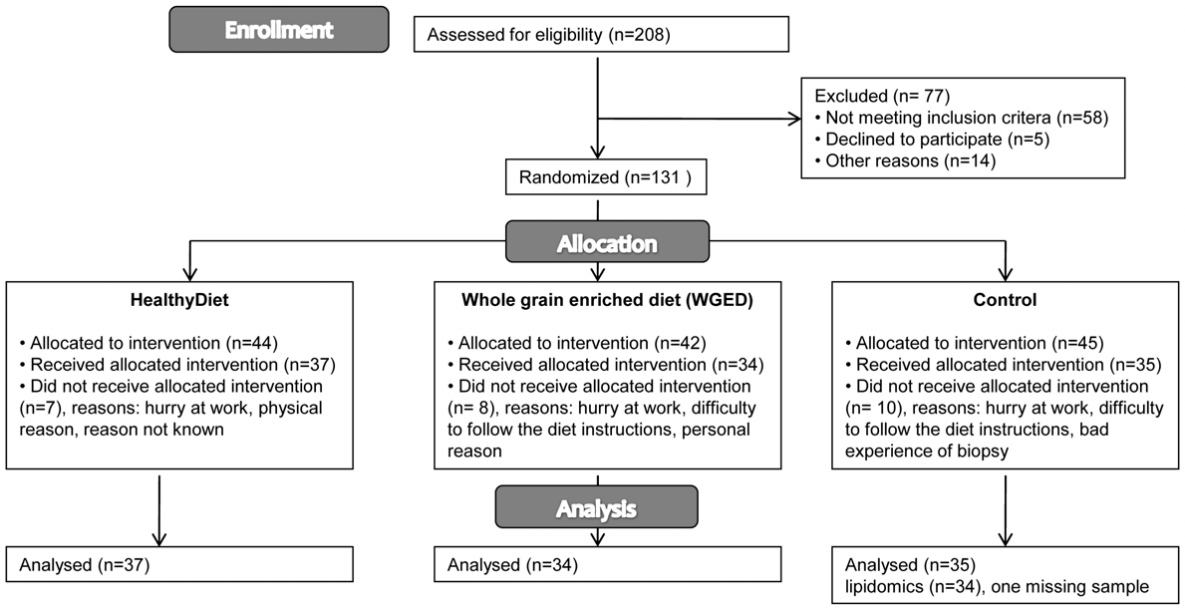
Flow diagram of the study.

Altogether 131 participants were recruited for a 12-week parallel controlled dietary intervention study from Kuopio area (**Figure 1**). The inclusion criteria were age 40–70 years, impaired glucose metabolism (fP-gluc 5.6–6.9 mmol/l (IFG) or in oral glucose tolerance test (OGTT) 2 hour P-gluc 7.8–11.0 mmol/l (IGT)) and at least two of the following: BMI 26–39 kg/m^2^, waist circumference ≥102 cm in men and ≥88 cm in women, serum triglycerides ≥1.7 mmol/l, HDL<1.0 mmol/l in men and <1.3 mmol/l in women or blood pressure ≥130/≥85 mmHg or use of medication for hypertension. The cut-off values were based on NCEP Adult Treatment Panel III, 2001. The participants were enrolled in the study sequentially. Of all participants, 86% had at least one medication. In the HealthyDiet group, 5.4% of the participants did not have any medication. The respective proportions were 14.7% and 22.9% in the whole grain enriched diet (WGED) group and Control group (χ^2^-test, p = 0.1). Most commonly used medications were statins (27%), angiotensin II inhibitors (27%), beta blockers (23%), calcium antagonists (20%), anticoagulants (17%) and ACE inhibitors (15%). None of the subjects used glucose lowering drugs and uses of statins, beta blockers or diuretics, and hormonal replacement therapy were very similar between the groups (Table 1). Besides some minor exceptions, participants continued their medications unchanged throughout the study.

**Table 1:** Baseline characteristics of the participants.^1^^,^ 2

	HealthyDiet *n* = 37	WGED^3^ *n* = 34	Control *n* = 35
**Gender, ** ***male/female***	17/20	17/17	18/17
**Age, ** ***years***	58±7	58±8	59±7
**Body weight, ** ***kg***	89.8±12.2	89.2±15.3	89.7±13.0
**Body mass index, ** ***kg/m^2^***	31.1±3.6	31.4±3.4	31.0±3.6
**Waist circumference, ** ***cm***	105.6±9.5	106.3±11.1	105.8±10.0
**Serum cholesterol, ** ***mmol/l***	5.1±0.9	5.1±1.0	5.4±1.0
**LDL cholesterol, ** ***mmol/l***	3.1±0.7	3.2±0.8	3.4±0.8
**HDL cholesterol, ** ***mmol/l***	1.3±0.3	1.2±0.4	1.3±0.3
**Serum triacylglycerols, ** ***mmol/l***	1.6±0.6	1.5±0.8	1.5±0.8
**Fasting plasma glucose, ** ***mmol/l***	6.1±0.5	6.1±0.4	6.2±0.5
**Plasma glucose OGTT 2 hour, ** ***mmol/l***	6.7±1.7	6.6±1.6	6.8±1.9
**Systolic blood pressure, ** ***mmHg***	137±13	135±16	139±12
**Diastolic blood pressure, ** ***mmHg***	89±7	86±8	88±7
**How many of the MetS characteristics fulfilled:** 4			
**2 characteristics, ** ***%***	11	15	14
***3*** ** characteristics, ** ***%***	43	38	43
***4*** ** characteristics, ** ***%***	24	29	29
**5 characteristics, ** ***%***	22	18	14
**Use of statins, ** ***n***	10	10	9
**Use of hormonal replacement therapy, ** ***n***	3	4	3
**Use of beta blocker or diuretics, ** ***n***	11	12	9

1 Values are mean ± SD,

2 There were no significant differences in clinical characteristics between the groups at baseline,

3 Whole grain enriched diet,

4 On top of the impaired fasting glucose or glucose tolerance.

The participants were randomly assigned by the study nurse to one of the following groups: HealthyDiet, WGED or Control. Randomization was performed by matching according to gender and medians of age, BMI and fasting plasma glucose of the study population at screening. The matching produced equal amounts of the certain strata classes among the groups. Altogether 106 participants completed the study. Majority of the drop-outs did not state the reason for drop-out. The reported reasons were busy at work, difficulty to follow the diet instructions, bad experience of biopsy and other physical or personal reasons. Drop-outs were significantly younger than completers (mean age was 55±8 vs. 59±7), but otherwise did not differ based on their clinical characteristics.

In the HealthyDiet group (*n* = 37) the participants replaced their habitually used cereal products with breads having a low postprandial glucose and insulin response, contributing up to 20–25% of total energy intake (40% share of endosperm rye bread, 10% share of sourdough whole meal wheat bread, and 50% share of a selection of commercial rye breads). The recommended intake of whole meal pasta was at least 3.5 deciliter (uncooked) a week. The fiber content (g/100 g) of the study cereal products was: endosperm rye bread 6.9, whole meal wheat bread 6.4, commercial rye breads 10–14.4 and pasta 6. Besides the above mentioned cereal products, one portion of their habitually used cereal product was allowed to be eaten daily, e.g. porridge, cereals or pastries. Participants were also instructed to eat fatty fish meals (100–150 g of fish/meal) three times a week. The following fish species were recommended: salmon, rainbow trout, bream, Baltic herring, roach, vendace, white fish, char, trout, red-fish, mackerel and anchovy. In fish preparation, participants were advised to avoid sources of saturated fat, such as butter and cream. Bilberries were eaten 3 portions per day. Bilberries were served as frozen, puree and dried powder. The total amount of three portions is equivalent to 300 grams of fresh bilberries. Habitual use of other berries was allowed with a max 3–4 portion a week.

In the WGED group (*n* = 34), the participants consumed the same cereal products as in the HealthyDiet group. Additionally, they were given whole grain oat biscuits that they were allowed to consume one portion per day on a voluntary basis. Biscuits contained 8–8.5 g/100 g of dietary fiber and 16–18 g/100 g of fat, of which 4.3–7.7 g was saturated. Participants in the WGED group were asked not to change their fish and berry consumption.

In the Control group (*n* = 35) participants replaced their habitually used breads with refined wheat breads (dietary fiber 3–4.3 g/100 g) and other cereal products, *e.g.* porridge or pasta, with low fiber products (<6 g/100 g dietary fiber). Participants were allowed to eat maximum of 1–2 portions of rye products per day. The intake of bilberries was not allowed and other berries were allowed maximum of 3–4 times per week with maximum of 1 deciliter at a time. Fish was allowed to be eaten no more than once a week. Otherwise, the habitual diet and living habits were kept unchanged in all groups.

Participants recorded daily the use of the test breads (all groups), pasta (HealthyDiet and WGED), oat biscuits (WGED), bilberries (HealthyDiet), and fish (HealthyDiet). Four-day food records (consecutive days) that included one weekend day were kept by the study persons during the run-in period and three times during the intervention period in all groups.

### Two-hour oral-glucose-tolerance test

OGTT was performed at baseline and after the 12-week intervention. After the fasting blood sample was drawn, the participants drank a glucose solution (75 g glucose/3 dl) within 3 min. Blood samples were drawn through the catheter 30, 60, 120 min after the beginning of the glucose solute ingestion for the measurement of plasma glucose and insulin concentrations. The glucose and insulin areas under the curve (AUCs) were calculated.

Disposition index (DI) is a product of measures of insulin sensitivity and first-phase insulin secretion which is predictive of conversion to diabetes [28], [29]. Insulinogenic index (IGI) and quantitative insulin sensitivity check index (QUICKI) were calculated as following: IGI = (insulin 30 min − insulin 0 min, pmol/L)/(glucose 30 min − glucose 0 min, mmol/L), and QUICKI = 1/(lg10(insulin 0 min, mU/L)+lg10(glucose 0 min, mg/dl)). Disposition index was calculated as a product of IGI and QUICKI. Homeostasis model of insulin resistance (HOMA-IR) was calculated as following: (fasting glucose mmol/l×fasting insulin mU/L)/22.5.

### Biochemical analyses

Concentrations of serum total, LDL and HDL cholesterol and triglycerides were analyzed using commercial kits (981813, 981656, 981823 and 981786, respectively, Thermo Electron Corporation, Vantaa, Finland) and Thermo Fisher Konelab 20XTi Analyzer (Thermo Electron Corporation, Vantaa, Finland). Plasma insulin concentration was analyzed with a chemiluminescent immunoassay (Advia Centaur Immunoassay System, Siemens Medical Solution Diagnostics,Tarrytown, NY, USA). Plasma glucose was analyzed by using Konelab 20XTi Clinical Chemistry Analyzer and Enzymatic photometric (glucose hexokinase) method (Konelab System Reagents, Thermo Fisher Scientific, Vantaa, Finland).

### Fatty acids

Fasting blood samples for fatty acids and lipidomics analyses were taken before and after the intervention. Both samples were available from 105 participants (HealthyDiet group *n* = 37, WGED group *n* = 34, Control group *n* = 34). Lipids from plasma samples (50 µl) were extracted by a mixture of chloroform and methanol (2∶1, 400 µl) after addition of 40 µl NaCl 0.9% (0.15 M) and 10 µl internal standard (heptadecantrienoate TG C17∶0 1659.24 mg/l+FFA C17∶0 584.1 mg/l). The mixture was vortexed 1 min and incubated 30 min at room temperature. The lower layer was separated (centrifuged 10 000 rpm 5 min) and evaporated into dryness under nitrogen flow.

The residue was dissolved into petroleumether (700 µl, boiling point 40–60° C) and vortexed. Bound fatty acids were transmethylated with NaOME (250 µl, sodium methoxide in dry methanol 0.5 M) by vortexing and boiling at 45° C for 5 minutes. The mixture was acidified by adding 15% solution of NaHSO_4_ (500 µl) and evaporated petroleumether was substituted by adding 200 µl of it (vortexed). The mixture was transferred into microtubes (glass tubes washed with 200 µl of petroleum ether) and centrifuged 10 000 rpm, 5 min. The petroleumether layer containing FAME and FFA was separated into a GC vial, evaporated under nitrogen flow and redissolved into hexane (500 µl) and vortexed.

1 µl aliquots were used for GC injection at 260° C (splitless). The Agilent 7890 Gas Chromatography equipped with HP-FFAP Polyethylene Glycol TP column (25 m×200 µm×0.3 µm) was used. H_2_ was used as carrier gas at a total flow of 44.5 ml/min. The initial oven temperature was 70° C for 1.5 min and the temperature was increased at rate of 15° C/min until 240° C. The fatty acids were detected by flame ionization detector at 300° C.

### Lipidomics

Plasma samples (10 µl) were diluted with 10 µl sodium chloride (0,9%) and 20 µl of an internal standard mixture containing 10 lipid classes and 100 µl chloroform/methanol (2∶1) were added. The mixture was homogenised by vortexing 2 minutes and the extraction time was around 40 minutes at room temperature. After centrifugation (10000 RPM, 3 minutes), an exact aliquot (60 µl) of the lower layer was transferred into an HPLC vial insert and 10 µl an external standard mixture containing 3 labelled standards was added to the lipid extract. The sample order for LC/MS analysis was randomised.

Lipid extracts were analyzed on a Waters Q-Tof Premier mass spectrometer combined with ultra performance liquid chromatography (UPLC). The column was an Acquity UPLC™ BEH C18 2.1×100 mm with 1.7 µm particles. Column temperature was kept at 50°C and the temperature of the sample organizer was set at 10°C. The binary solvent system consisted of A. water (1% 1M NH_4_Ac, 0.1% HCOOH) and B. LC-MS grade isopropanol∶acetonitrile (1∶1, 1% 1M NH_4_Ac, 0.1% HCOOH). The gradient started from 65% A/35% B and reached 80% B in 2 min, 100% B in 7 minutes and remained at this level for next 7 minutes. The total run time including a 4 min re-equilibration step was 18 min. The flow rate was 400 µl/min and the injection volume 2.0 µl.

The data was collected in centroid form by using electro spray ionization + mode at mass range of m/z 300–1200 and with scan duration of 0.2 sec. The voltages of the sampling cone and capillary were 40.0 V and 3.0 kV, respectively. The source temperature was set at 120°C and nitrogen was used as desolvation gas (795 L/h) at 270°C. Reserpine (200 µg/L) was the lock spray reference compound at a flow rate of 8 µl/min and the scan was done at 10 s frequency.

The data was processed by using MZmine 2 software [30] and the lipid identification was based on an internal spectral library. The MZmine 2 data processing included chromatogram building, peak deconvolution, deisotoping, alignment, filtering and gap filling. The quality of data was first estimated by checking the shifts in retention times and peaks in the blank control samples. There were the maximum of 0.02 minute shifts in the retention times of standard compounds and only a few peaks were documented in the blanks.

Lipids were identified by comparing with an internal spectral library including MS and MS/MS data. All monoacyl lipids except cholesteryl esters, such as monoacylglycerols and monoacyl-glycerophospholipids, were normalized with PC(17∶0/0∶0), all diacyl lipids except ethanolamine phospholipids with PC(17∶0/17∶0), all ceramides with Cer(d18∶1/17∶0), all ethanolamine phospholipids with PE(17∶0/17∶0), and all TG and cholesteryl esters with TG(17∶0/17∶0/17∶0). Unidentified lipids were calibrated with PC(17∶0/0∶0) for retention times less than 300 seconds, with PC(17∶0/17∶0) for retention times between 300 and 410 seconds, and with TG(17∶0/17∶0/17∶0) for higher retention times. Identifications and exact fatty acid compositions of all of the most interesting lipids were confirmed with MS/MS analyses.

### Statistical analyses

The primary aim of this study was to see changes in glucose metabolism. Power calculations were based on fasting glucose. By using test significance α-level 0.05, 80% power and aim to see 5% difference between the groups the appropriate sample size was 37/group. Statistical analyses were performed using the SPSS statistical software (version 14.0, SPSS Inc., Chicago, IL) and R Project for Statistical Computing version 2.7.2 [31] and nlme R-package version 3.1–96 [32]. The normality of the distributions of the variables was estimated based on histograms. Linear mixed-effect models were used to analyze group differences in the baseline and during the intervention. Quantitative dependent variables were transformed to base-10 logarithmic scale to account for non-normal distributions. Selected confounding phenotypes were included in the models as covariates (age, gender, BMI, insulin and glucose), and participant's identifiers were included as a grouping random effect. Interaction term between group and intervention time-point (before or after intervention) was used to examine group related changes during the intervention. The Control group was used as a reference group when comparing group differences. Benjamini-Hochberg false discovery rate (FDR) was used to adjust results for multiple comparisons [33]. FDR *p*-value <0.05 was considered as statistically significant. The association of EPA and DHA with DI was tested using Spearman rank correlation and linear regression model (age, gender and weight changes were included in the model). The results from regression model need to be discussed with reservation, since the distributions of the variables were not normal. Kruskal-Wallis test was performed in order to test differences in changes in DI between different EPA and DHA quartiles.

## Results

### Clinical characteristics and dietary intake

There were no statistically significant differences between the groups at baseline (**Table 1**). The results of the basic clinical and biochemical parameters (apart from glucose and insulin) during the intervention periods will be reported elsewhere. Body weight remained constant during the study in all groups.

Self-reported compliance with the diets was good. The mean test bread consumption during the intervention period was 7.7, 7.9 and 6.8 portions per day in the HealthyDiet, WGED and Control groups, respectively. In the HealthyDiet group, the mean fish consumption was 3.3 fish meals per week. Mostly used fish species were salmon, rainbow trout, vendace and Baltic herring. The mean bilberry consumption in the HealthyDiet group was 3.2 portions per day.

**Figure 2:**
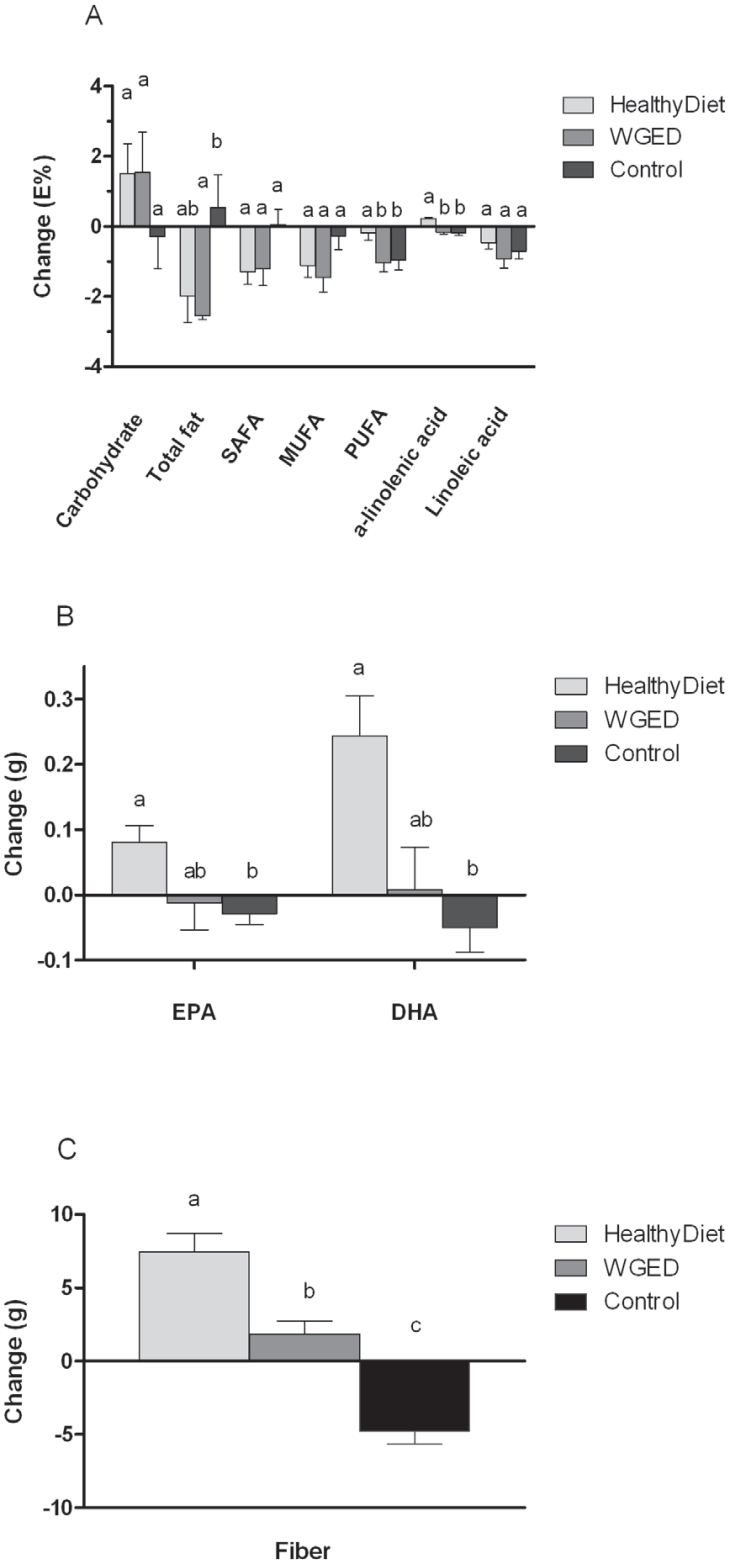
Changes in dietary intake during the 12-week intervention in the HealthyDiet (n = 36), Whole grain enriched diet (WGED) (n = 34) and Control (n = 35) groups. Bars represent mean of absolute changes ± SD. Means without a common letter differ in mixed model comparison, FDR *p*<0.05.

At the baseline, the intake of dietary fiber was higher in the HealthyDiet group than in the Control and WGED groups (FDR *p* = 0.013). Otherwise, dietary intake did not differ at baseline among the groups. Energy intake did not change during the intervention. The intake of EPA, DHA, α-linolenic acid and fiber increased in the HealthyDiet group compared with the Control group (**Figure 2**). In the WGED group, the intake of total fat decreased and fiber increased compared with the Control group. Within the Control group the intake of PUFA, EPA, DHA and fiber decreased during the intervention.

### Glucose and insulin metabolism

**Figure 3:**
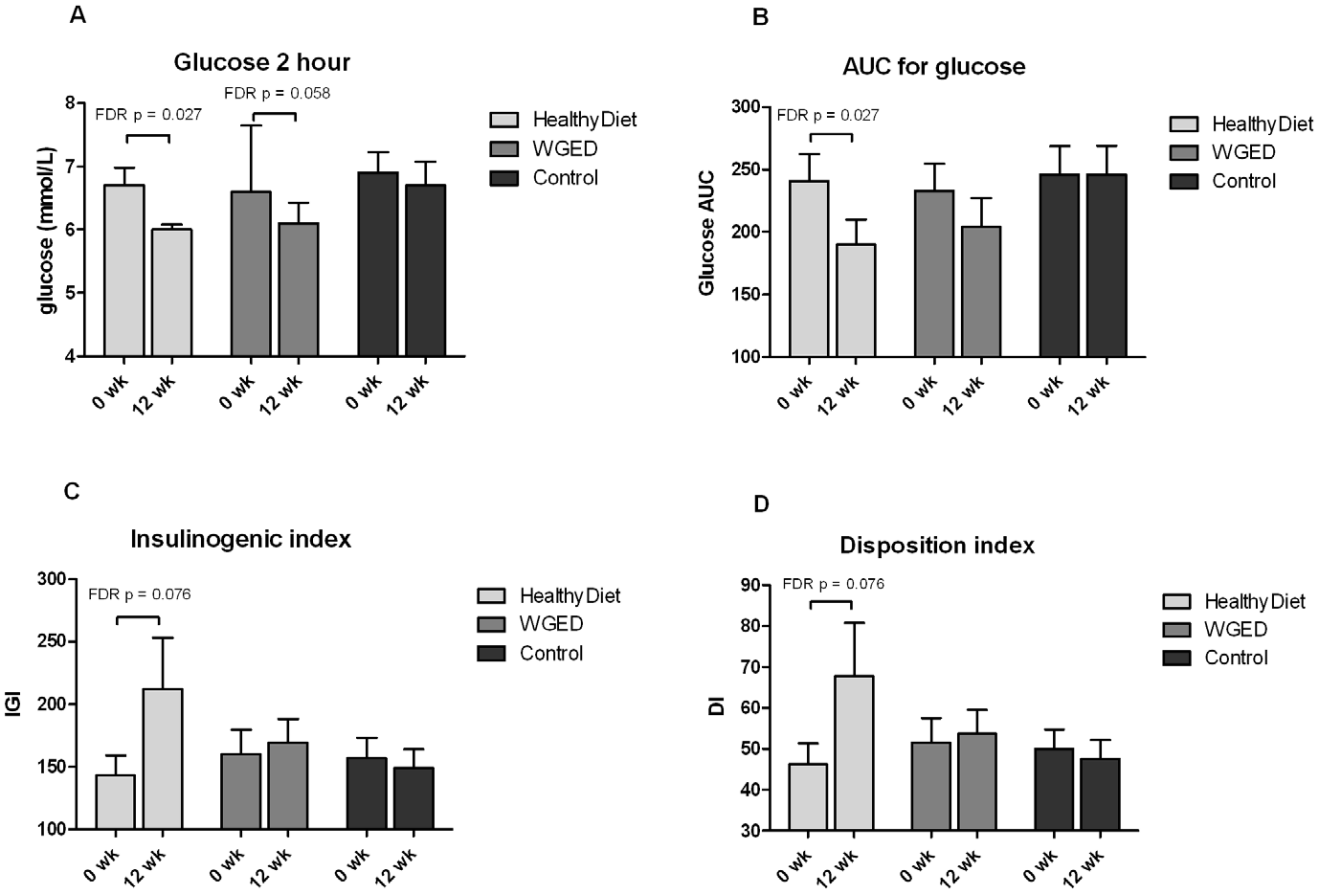
Bar charts of glucose metabolism parameters in each group before and after the intervention. A) plasma 2 hour glucose, B) area under the curve (AUC) for glucose, C) Insulinogenic index (IGI) and D) Disposition index (DI) in OGTT. Values are means ± SEM, *n* = 37, 34 and 35 in the HealthyDiet, Whole grain enriched diet (WGED) and Control groups, respectively, except for IGI and DI, *n* = 36 before and *n* = 35 after the intervention in the HealthyDiet group, and *n* = 32 before and after the intervention in the WGED group.

There were no statistical differences in changes of glucose and insulin metabolism between the groups (**Table 2**). In the HealthyDiet group, the within-the-group comparison revealed decreases in the 2-hour glucose concentration and AUC for glucose (Table 2, **Figure 3**). There was a trend towards improved IGI (*p* = 0.016 and FDR *p* = 0.076) and DI (*p* = 0.019 and FDR *p* = 0.076) within the HealthyDiet group (Table 2, Figure 3).

**Table 2:** Glucose and insulin parameters at baseline and after the 12-week intervention.^1^

	HealthyDiet *n* = 37				WGED^2^ *n* = 34				Control *n* = 35		
	0 wk	12 wk	FDR *p* group^3^	FDR *p* time* group^4^	0 wk	12 wk	FDR *p* group^3^	FDR *p* time* group^4^	0 wk	12 wk	FDR *p* group^3^
**Fasting glucose (mmol/L)**	6.1±0.5	6.0±0.5	0.71	0.91	6.1±0.4	6.1±0.5	0.99	0.90	6.2±0.5	6.2±0.5	0.81
**Glucose 2 h (mmol/L)**	6.7±1.7	6.0±0.5	**0.027**	0.53	6.6±1.6	6.1±1.9	0.058	0.68	6.9±1.9	6.7±2.2	0.81
**Fasting insulin (mU/L)**	11.7±5.9	12.5±6.3	0.37	0.91	12.0±6.2	13.7±8.0	0.16	0.73	12.8±6.6	13.2±6.3	0.81
**Insulin 2 h (mU/L)**	62.6±48.2	61.3±46.7	0.69	0.91	78.3±65.6	65.9±59.9	0.16	0.67	72.1±54.2	72.3±55.7	0.95
**AUC** 5 **for glucose (mmol/L)**	241±130	190±121	**0.027**	0.49	233±128	204±137	0.16	0.53	246±136	246±137	0.84
**AUC** 4 **for insulin (mU/L)**	6787±3279	7509±3772	0.64	0.91	7650±6004	7523±4740	0.57	0.68	7766±4173	7514±3861	0.89
**HOMA-IR** 6	3.2±1.8	3.4±1.9	0.43	0.91	3.3±1.7	3.7±2.3	0.16	0.73	3.6±2.0	3.7±1.9	0.89
**IGI** 7	143±99^9^	212±251^10^	0.076	0.49	160±115^11^	169±113^11^	0.99	0.91	157±97	149±89	0.89
**QUICKY** 8	0.33±0.02	0.33±0.02	0.37	0.91	0.33±0.02	0.32±0.03	0.16	0.90	0.32±0.02	0.32±0.02	0.81
**Disposition index**	46.3±30.4^9^	67.8±78.8^10^	0.08	0.49	51.5±35.2^11^	53.7±34.3^11^	0.99	0.91	50.0±28.2	47.6±27.2	0.82

1 Values are mean ± SD,

2 Whole grain enriched diet,

3 False discovery rate p-value for effect of time within the groups,

4 False discovery rate p-value for interaction of time and group (time*group),

5 Area under the curve in 2-hour OGTT,

6 Homeostasis model of insulin resistance,

7 Insulinogenic index,

8 Quantitative insulin sensitivity check index,

9 *n* = 36,

10 *n* = 35,

11 *n* = 32.

### Fatty acids and lipidomics

There were no differences in plasma fatty acids between the groups at baseline. Proportions of di-homo-γ-linolenic acid decreased and polyunsaturated long chain fatty acids (docosapentaenoic acid, EPA, DHA) increased in the HealthyDiet group compared with the Controls (**Table 3**). Within the Control group, DHA decreased during the intervention. There were no significant changes in plasma fatty acid composition in the WGED group.

**Table 3:** Plasma fatty acids at baseline and after the 12-week intervention.^1^

	HealthyDiet *n* = 37				WGED^2^ *n* = 34				Control *n* = 34		
Fatty acid, *%*	0 wk	12 wk	FDR *p* group^3^	FDR *p* time* group^4^	0 wk	12 wk	FDR *p* group3	FDR *p* time* group^4^	0 wk	12 wk	FDR *p* group^3^
**Myristic (14∶0)**	1.0±0.47	1.09±0.41	0.18	0.96	0.82±0.43	0.93±0.49	0.23	0.82	0.79±0.41	0.92±0.42	0.22
**Myristoleic (14∶1(n-5))**	0.10±0.04	0.10±0.05	0.89	0.92	0.10±0.05	0.10±0.05	0.64	0.96	0.10±0.05	0.10±0.04	0.83
**Pentadecanoic (15∶0)**	0.21±0.05	0.21±0.06	0.86	0.92	0.21±0.05	0.20±0.06	0.42	0.81	0.21±0.05	0.20±0.05	0.56
**Palmitic acid (16∶0)**	26.90±1.99	27.66±2.22	0.13	0.58	26.46±1.95	27.25±2.17	0.07	0.56	26.57±1.59	26.86±2.00	0.56
**Palmitoleic (16∶1(n-7))**	2.66±0.92	2.58±0.77	0.89	0.76	2.58±0.72	2.79±0.79	0.23	0.76	2.36±0.72	2.41±0.64	0.82
**Stearic (18∶0)**	9.08±0.90	9.42±0.95	**0.045**	0.76	8.93±0.98	8.94±1.19	0.64	0.57	8.95±0.85	9.14±1.00	0.35
**Oleic (18∶1(n-9))**	23.40±3.19	22.31±3.37	**0.009**	0.09	23.58±2.93	23.80±2.97	0.64	0.96	23.00±2.88	23.12±2.66	0.87
**Cis-vaccenic (18∶1(n-7))**	2.38±0.33	2.12±0.30	**7×10^−4^**	0.11	2.47±0.28	2.41±0.36	0.42	0.83	2.31±0.28	2.21±0.23	0.08
**Linoleic (18∶2(n-6))**	19.39±3.29	18.52±3.09	0.13	0.32	20.13±2.31	19.41±2.67	0.33	0.53	20.59±3.03	20.66±3.02	0.75
**γ-linolenic (18∶3(n-6))**	0.23±0.08	0.23±0.11	0.79	0.16	0.20±0.07	0.22±0.08	0.23	0.82	0.22±0.08	0.26±0.12	0.07
**α-linolenic (18∶3(n-3))**	0.96±0.26	1.10±0.23	**0.002**	0.09	1.02±0.36	0.94±0.35	0.23	0.46	1.00±0.34	1.00±0.26	0.88
**Di-homo-γ-linolenic (20∶3(n-6))**	1.79±0.32	1.61±0.35	**0.001**	**7×10^−4^**	1.79±0.45	1.77±0.44	0.90	0.46	1.78±0.41	1.85±0.32	0.26
**Aracidonic (20∶4(n-6))**	6.09±1.25	5.45±0.99	**0.004**	0.27	6.08±1.58	5.79±1.41	0.32	0.92	6.31±1.52	6.09±1.52	0.51
**EPA (20∶5(n-3))**	1.60±0.77	2.55±1.21	**9×10^−4^**	**7×10^−4^**	1.51±0.69	1.41±0.91	0.42	0.92	1.66±0.96	1.45±0.76	0.17
**DPA (22∶5(n-3))**	0.83±0.18	0.95±0.24	**0.001**	**1×10^−4^**	0.79±0.14	0.80±0.18	0.64	0.46	0.84±0.16	0.80±0.16	0.18
**DHA (22∶6(n-3))**	3.43±1.09	4.10±1.09	**7×10^−4^**	**5×10^−7^**	3.34±0.14	3.22±1.3	0.23	0.46	3.31±0.98	2.92±0.99	**0.009**

1 Values are mean ± SD, *n* = 105,

2 Whole grain enriched diet,

3 False discovery rate p-value for effect of time within the groups,

4 False discovery rate p-value for interaction of time and group (time*group).

A total of 364 lipids were identified and quantified by the UPLC-MS platform including lipid classes such as TGs, LPCs, phophatidylcholines (PCs), phosphatidylserines (PSs), phosphatidyl ethanolamines (PEs), sphingomyelins (SMs) and ceramides. There were no significant differences among the groups at baseline. Using the within-the-group comparisons, 152, 91 and 178 lipids changed significantly (FDR *p*<0.05) in the HealthyDiet, WGED and Control groups, respectively. Mixed model analysis revealed 25 significantly changed lipids when comparing the HealthyDiet and the WGED groups to the Control group (**Table 4**). All the significant changes occurred in the HealthyDiet group. Multiple TGs with the long chain PUFAs, including TG(60∶12), TG(60∶13), TG(58∶11), TG(56∶10), increased in the HealthyDiet group. Also LPC(20∶5), PC(36∶5), PC(38∶7e), PC(40∶7e) and TG(55∶7) increased, while the PS(38∶2) and LPC(20∶3) decreased. LPC(22∶6) increased within the HealthyDiet group (FDR *p* = 0.005), but in a comparison of all three groups together this increase did not reach the level of statistical significance (FDR *p* = 0.088). Odd chain TGs, except TG(55∶7), or ceramides did not change during the intervention.

**Table 4:** Lipids that change significantly different between the groups after the 12-week intervention period.^1^

Lipid	Beta HD^2^ vs. Control	FDR *p* HD^2^ vs. Control^3^^,^ 4	Beta HD^4^	FDR *p* HD^2^^,^ 4 ^,^ 5	Beta WGED^6^	FDR *p* WGED^4^^,^ 5 ^,^ 6	Beta Control	FDR *p* Control^4^^,^ 5	Beta HD^2^ vs. WGED^6^	FDR *p* HD^2^ vs. WGED^6^^,^ 7
**TG(60∶13)**	0.35	**0.007**	0.35	**7×10^−6^**	0.06	0.43	−0.01	0.87	0.27	0.08
**TG(60∶12)**	0.46	**3×10^−4^**	0.42	**3×10^−6^**	0.07	0.37	−0.05	0.49	0.33	0.07
**TG(60∶12)** 8	0.43	**5×10^−4^**	0.41	**5×10^−6^**	0.08	0.27	−0.03	0.66	0.31	0.07
**TG(58∶11)**	0.33	**0.008**	0.37	**4×10^−6^**	0.08	0.33	0.04	0.50	0.27	0.08
**TG(58∶11)** 8	0.29	**0.013**	0.34	**4×10^−6^**	0.06	0.43	0.05	0.38	0.26	0.08
**TG(58∶10)**	0.24	**0.026**	0.30	**4×10^−6^**	0.05	0.42	0.06	0.29	0.23	0.08
**TG(58∶10)** 8	0.22	**0.041**	0.31	**4×10^−6^**	0.08	0.23	0.08	0.18	0.21	0.08
**TG(56∶10)**	0.24	**0.013**	0.31	**4×10^−6^**	0.07	0.31	0.05	0.26	0.22	0.08
**TG(56∶9)**	0.22	**0.041**	0.31	**4×10^−6^**	0.06	0.38	0.09	0.14	0.24	0.07
**TG(56∶6)**	0.17	**0.019**	0.18	**6×10^−6^**	0.04	0.44	−0.001	0.97	0.13	0.12
**TG(55∶7)**	0.27	**0.036**	0.29	**7×10^−5^**	0.10	0.17	0.01	0.87	0.19	0.19
**TG(54∶8)**	0.25	**0.020**	0.31	**5×10^−6^**	0.08	0.24	0.05	0.40	0.21	0.08
**PC(40∶7e)**	0.14	**0.013**	0.11	**3×10^−4^**	0.04	0.31	−0.03	0.25	0.06	0.35
**PC(40∶7e)** 8	−0.15	**0.041**	−0.03	0.48	0.09	**0.044**	0.12	**0.007**	−0.13	0.08
**PC(40∶4)**	−0.23	**0.040**	−0.04	0.58	0.12	0.07	0.18	**0.004**	−0.18	0.17
**PC(38∶7e)**	0.13	**0.013**	0.11	**2×10^−4^**	0.02	0.63	−0.02	0.39	0.08	0.19
**PC(38∶7)**	0.15	**0.040**	0.19	**1×10^−5^**	0.08	0.08	0.03	0.49	0.10	0.22
**PC(38∶4e)**	−0.14	**0.013**	−0.03	0.33	0.03	0.22	0.10	**0.004**	−0.07	0.24
**PC(36∶5)**	0.19	**0.013**	0.24	**4×10^−6^**	0.07	0.13	0.04	0.41	0.16	0.08
**PE(40∶7)**	−0.13	**0.040**	−0.08	**0.032**	0.04	0.24	0.06	0.10	−0.12	0.08
**PE(38∶7e)**	0.20	**0.013**	0.20	2**×**10^−5^	0.08	0.09	−0.01	0.87	0.11	0.22
**PE(38∶4)/PC(35∶4)**	−0.13	**0.013**	−0.02	0.43	0.06	**0.048**	0.10	**0.009**	−0.09	0.08
**PS(38∶2)**	−0.14	**0.013**	−0.09	**0.010**	0.02	0.54	0.05	0.10	−0.11	0.08
**LPC(20∶5)**	0.21	**0.007**	0.15	**0.002**	−0.001	0.97	−0.05	0.17	0.16	0.08

1 *n* = 105,

2 HealthyDiet group,

3 False discovery rate p-value for interaction of time and group (time*group), the HealthyDiet group compared with the Controls,

4 Age, gender, BMI, Insulin and glucose are used as covariates,

5 False discovery rate p-value for effect of time within the groups,

6 Whole grain enriched diet group,

7 False discovery rate p-value for interaction of time and group, the HealthyDiet group compared with the WGED group,

8 Lipids which are mentioned twice are isobaric, meaning that the fatty acid composition differs.

### Association between plasma EPA and DHA and glucose metabolism

**Figure 4:**
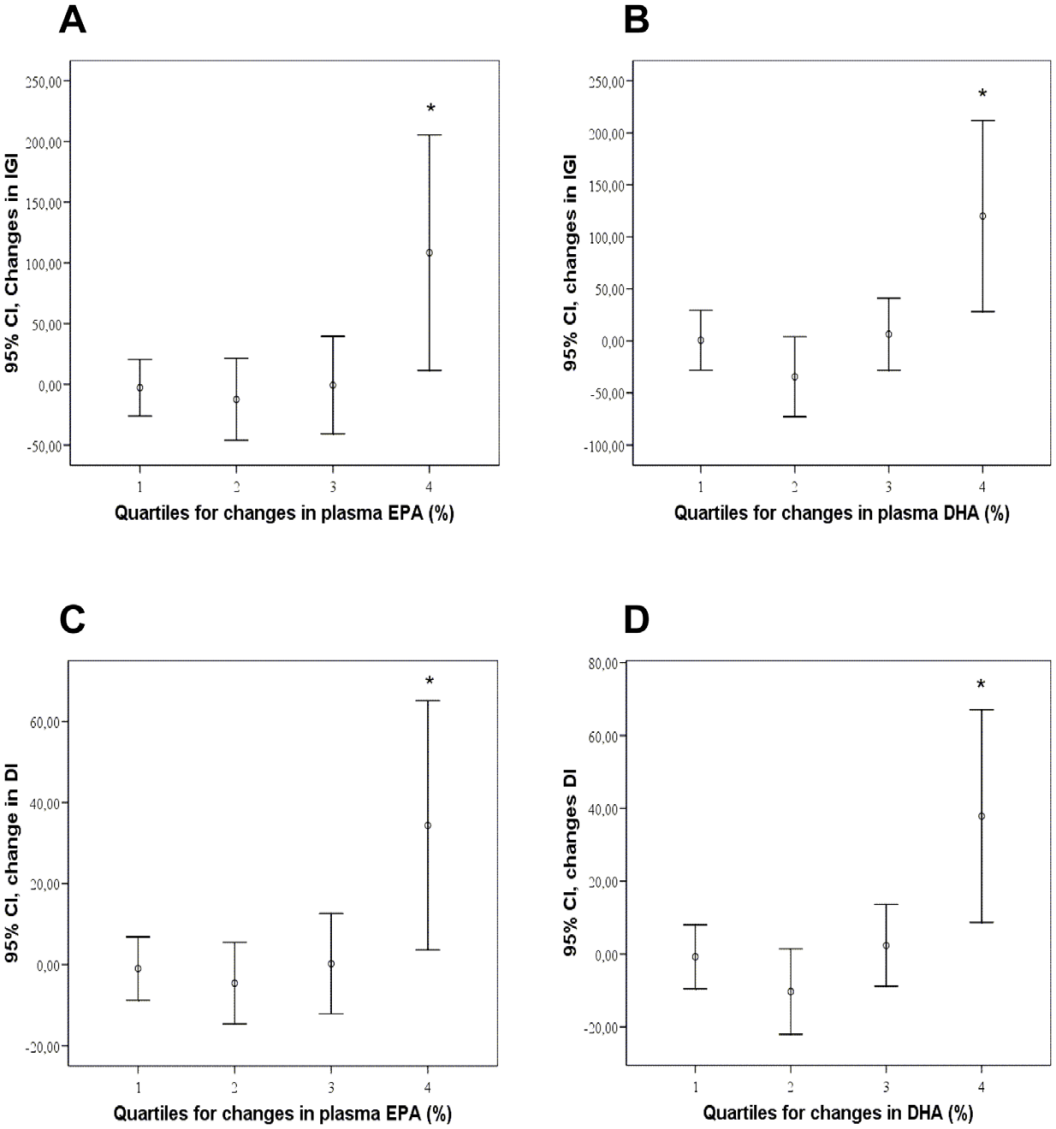
Associations between glucose metabolism and plasma EPA and DHA content. Changes in insulinogenic index (IGI) (A and B) and disposition index (DI) (C and D) during the intervention period (95% Confidence interval) according to quartiles of changes in plasma EPA (A and C) and DHA (B and D) content (%). * *p*<0.01 in Kruskal-Wallis test. All groups combined, *n* = 99. Ranges of changes in EPA quartiles: 1 = −2.3–0.4% units; 2 = −0.3−0.1% units; 3 = 0.1−0.6% units; 4 = 0.6−4.2% units, and in DHA quartiles: 1 = −2.4–0.6% units; 2 = −0.6–0.1% units; 3 = −0.04−0.7% units; 4 = 0.7−3.0% units.

Regression model revealed significant association between the changes in plasma EPA and DHA with changes in IGI (*R* = 0.366, *p* = 0.009 and *R* = 0.379, *p* = 0.006) and DI (*R* = 0.366, *p* = 0.009 and *R* = 0.382, *p* = 0.005, respectively). The inclusion of changes in the fiber intake in the regression model did not alter associations. There were also positive correlations between the changes in EPA and DHA and changes in IGI (*r* = 0.309, *p* = 0.002 and *r* = 0.413, *p*<0.001, respectively) and DI (*r* = 0.313, *p* = 0.002 and *r* = 0.341, *p* = 0.001, respectively). IGI and DI improved most in the highest quartiles of changes in EPA and DHA during the 12-week intervention (**Figure 4**).

## Discussion

We studied the effects of whole grain and low insulin response grain products, fatty fish, and bilberries on glucose metabolism, plasma fatty acids and lipidomic profile in individuals with features of the metabolic syndrome. We found that diet with high intake of whole grain and low insulin response grain products, fatty fish and bilberries (HealthyDiet) appeared to improve glucose metabolism and altered plasma lipidomic profile markedly, while exclusive carbohydrate modification caused only minor changes. Interestingly, we identified an association between the increases in plasma EPA and DHA contents and improvement in glucose metabolism.

A decreased AUC for glucose and a trend towards improved IGI and DI in the HealthyDiet group suggest that the intake of whole grain and low insulin response grain products, fatty fish and bilberries had positive effects on insulin secretion and glucose disposal in this study. There was also a trend towards improved 2-hour glucose, but not for IGI and DI, in the WGED group. The results for IGI and DI were very similar, which is not surprising as DI is calculated from IGI. In the entire study group, a higher increase in EPA and DHA was curvilinearly associated with IGI and DI and thus to improved glucose metabolism. Besides fatty fish, bilberries may also have had a role in the improved glucose metabolism, but we do not have a biomarker for bilberry intake which would have enabled better estimation of its effects. However, not all subjects in the 4^th^ quartile of EPA and DHA changes were in the HealthyDiet group, which suggests that changes in the EPA and DHA may have an independent association with the glucose metabolism. Recently, Stull and colleagues [34] showed that insulin sensitivity was enhanced after 6 wk dietary supplementation with bioactive substances from bilberries in 32 obese, insulin-resistant persons.

Results from the intervention studies regarding the effects of (n-3) PUFA of animal origin on glucose metabolism (mostly insulin resistance) have been variable and most of the studies have been performed with fish oil supplements [18]. There are multiple discrepancies between the (n-3) PUFA intervention studies relating to e.g. methods they have used to assess glucose metabolism, health status of participants, diet or duration of the study [18]. Improvements in insulin resistance have not been observed in healthy individuals or diabetic persons, but the positive effects have been identified in obese individuals [16], [18], [35]. Our study population could have been an optimal target group for the application of dietary strategy to improve glucose metabolism, since the participants had IFG or IGT, but not diabetes. However, the diet rich in whole grain and low insulin response grain products, bilberries and fatty fish did not alter insulin resistance but instead improved insulin secretion in this study. Associations between the changes in plasma EPA and DHA content and improved IGI and DI were curvilinear (Figure 4), meaning that the improved insulin secretion and glucose disposal existed only in the highest quartiles of changes in EPA and DHA. This may partly explain the discrepancy on the evidence related to (n-3) PUFA of animal origin and glucose metabolism. Future studies are needed to confirm these results and to find the optimal amount of fish needed to achieve improvement in insulin metabolism in subjects with IFG or IGT.

High-carbohydrate-low-GI diet has been shown to improve DI in persons with impaired glucose tolerance [2], [36] or type 2 diabetes [37]. In two studies the duration of the low-GI diet has been 4 to 12 months until the increase in DI has became evident [36], [37]. However, Laaksonen and colleagues detected improved DI after 12-week consumption of carbohydrate modified diet with cereal products replaced with rye-based products and whole-grain pasta [2]. It is unlikely, but still possible, that our intervention period of 12-weeks was too short to observe the improvement in DI in the WGED group, while the effects of (n-3) PUFA appeared earlier. In addition, the improvement in DI in the HealthyDiet group might have reached the level of statistical significance after the longer intervention period. Moreover, there were slight differences in the cereal diets between WGED group and in the study of Laaksonen *et al* [2] that might have had an impact on the differences found.

Fatty acid analyses revealed increases in the plasma content of long chain (n-3) PUFAs in the HealthyDiet group, whereas in the Control group plasma content of DHA decreased without a change in the WGED group. Furthermore, lipidomics analysis revealed increases in TGs with long chain (n-3) PUFAs in the HealthyDiet group. These changes confirm the good compliance with the instructions of fish intake. Interestingly, we detected increases in highly unsaturated TGs in the HealthyDiet group (*e.g.* TG(60∶12) and TG(60∶13)), which we have not identified in our previous studies.

We observed increased plasma concentrations of LPC species in the HealthyDiet group. LPCs are linked with atherosclerosis by virtue of their effects on arterial wall, smooth muscle cells and macrophages, although also other putative antiatherogenic effects have been demonstrated [38]. LPC is a major component of oxidized LDL [38], [39]. In high density lipoprotein (HDL) fraction, it is more abundant in persons with high HDL cholesterol [40]. The acyl chains in LPC and lysophosphatidicacid (LPA) molecules vary from saturated to highly unsaturated, and from 14 to 22 carbons, and their biological functions may vary depending on the acyl chain [38], [41], [42]. Block and colleagues [42] demonstrated that dietary EPA and DHA fatty acids are incorporated into plasma LPCs, but not into LPAs. Here we found increase in LPC(20∶5) after a 12-week consumption of fatty fish. LPC(22∶6) also increased within the HealthyDiet group, but among the groups this increase did not reach the level of statistical significance. The ability to increase LPC(20∶5) and LPC(22∶6) with dietary ingestion of fatty fish may be important, since LPC are believed to be major carriers of DHA to the brain [43].

We found an increase of odd chain triglyceride TG(55∶7) in the HealthyDiet group. Other odd chain TG:s did not change. Odd chain fatty acids 15∶0 and 17∶0 have been considered as a biomarker of dairy fat intake, and thus even hypothesized to have protective effects against heart disease, stroke and insulin resistance [44]–[47]. These observations have been based on plasma and adipose tissue levels of 15∶0 and 17∶0 rather than dietary recording methods. Contradictory, association between the intake of dairy fat and relative serum content of 17∶0 have not been clear in every study [48] and also inverse associations have been observed [49]. Furthermore, in the large cohort study EPIC, there was a strong positive correlation (r = 0.8, p≤0.01) between the total intake of fish and plasma content of 17∶0 [50]. In our study, plasma 15∶0 did not change during the intervention, and 17∶0 was used as an internal standard in the fatty acid analyses, so we could not determine its plasma levels. However, TGs with the odd number of carbons most likely include 15∶0 and17∶0 fatty acids. Based on 4-day food diaries dietary intake of SAFA decreased from 12 E% to 10.7 E% within the HealthyDiet group. This indicates compliance to the instruction to avoid cream and butter in fish preparation. Decreased dairy fat intake should also have been shown in a decrease of odd chain fatty acids, if those could be considered as good biomarkers for dairy fat intake. Fatty acid 17∶0 is present in the fat of fish in low amounts (0.31–2.0% depending on fish species) [51], [52]. Salmon contains around 40 mg of 17∶0 and 20 mg of 15∶0 fatty acids per 100 g of fish [53]. Our results suggest that intake of odd chain fatty acids from dairy fat decreased, but instead the same amount of it came from fatty fish, keeping the plasma concentration constant. Therefore, we believe that it is questionable to consider odd chain fatty acids as biomarkers of dairy fat intake and the associated health effects might as well be related to high intake of fish. Further studies are needed to address this issue.

In the WGED group, we did not find significant changes in lipids in comparison with the Control group. This is consistent with our earlier findings, *i.e.*, the intake of low insulin response grain products (rye bread and pasta) [12] or the intake of high-fiber rye bread [54] did not lead to changes in serum lipidomic profiles. Changes in the HealthyDiet group are different than those which we found in our previous study, where the study persons ate at least 4 fatty or lean fish meals per week [25]. In that study, unlike in the present one, we found a decrease in plasma ceramides and LPCs as a result of fatty fish diet. However, participants in that study were coronary heart disease patients with multiple medications, which may have affected the results. This discrepancy also suggests that even if carbohydrate modification itself does not markedly change serum lipidomic profile, it has effects with bilberries and fatty fish, which cause changes in lipidomic profile. On the other hand, it is possible that the intake of three fish meals per week was not enough to see all the effects, which were detectable after four fish meals per week. Furthermore, the power calculations were based on fasting glucose, and it is possible that there was not enough power to see all changes in lipidomics or other glucose parameters. To see also the individual effects of fish or bilberries, and also synergistic effects of grain products, fish and bilberries, separate intervention groups with increased intake of only fish or only bilberries would have been needed.

In conclusion, our results suggest that diet rich in whole grain and low insulin response grain products, bilberries, and fatty fish alter plasma lipidomic profiles and may be associated with improved glucose metabolism. Therefore, in long-term these dietary components may have a beneficial role in the prevention of type 2 diabetes in persons with impaired glucose metabolism.

## Supporting Information

Checklist S1 **CONSORT checklist.**
(DOC)

Protocol S1 **Trial protocol.**
(DOC)
