# Supplementary material for: Whole Grain Products, Fish and Bilberries Alter Glucose and Lipid Metabolism in a Randomized, Controlled Trial: The Sysdimet Study
Source: PLoS One. 2011 Aug 25;6(8):e22646. doi: 10.1371/journal.pone.0022646 (PMC3161986; doi:10.1371/journal.pone.0022646)
Supplement: Protocol S1 — Trial protocol. (DOC) [file pone.0022646.s002.doc]

**PROTOCOL S1**

**1. ABSTRACT**

**Applicant:** Matti Uusitupa, M.D., Dr. Med. Sci, Professor

**Research group:** Ursula Schwab, PhD, Docent, Marjukka Kolehmainen, PhD, Leena Pulkkinen,

PhD, Docent, David Laaksonen, MD, PhD, MPH, Docent., Kaisa Poutanen, DSc (Tech), Research Professor

**Co-applicant within consortium:** Matej Oresic, PhD, Docent

**International collaborators:** Sander Kersten, PhD, Professor, Gabriele Riccardi, MD, Professor

***Systems biology approach to understand dietary modulation of gene expression and metabolic pathways in subjects with abnormal glucose metabolism (Sysdimet)***

**Amount of funding applied for:** 621 500 €

**Number of person years of work and funding period:** 9.5 person years; 1.1.2007-31.12.2010

**Sites of research:** Department of Clinical Nutrition, Food and Health Research Centre, University of Kuopio, Kuopio; VTT Technical Research Centre, Espoo, Finland; Wageningen University and Reseach Centre, Wageningen, The Netherlands; University of Naples, Italy

**Objective and brief description of the project:** The metabolic syndrome (MS) and type 2 diabetes (T2DM) are the most important health problems worldwide. In Finland the prevalence of T2DM is 12-15% among middle-aged people. The prevalence of less marked disturbances in glucose metabolism and MS is 30-40%. Because MS and T2DM are important risk factors for cardiovascular diseases (CVD), the leading cause of death in western countries, all efforts to reverse the epidemic increase in the incidence of MS and T2DM are warranted. We have focused for years on the prevention and non-pharmacological treatment of T2DM and its complications including studies on genetic regulation of glucose and lipid metabolism after dietary modifications. In our recent projects, we have studied the effects of long-term dietary interventions on gene expression profiles of fat tissue in subjects who are at risk of T2DM. The ultimate goal of these projects has been to identify genes and gene clusters and their biological pathways that respond to dietary modification and modulate glucose and lipid metabolism, and to develop dietary strategies for prevention of T2DM.

The main goal of this project is to find nutrition related early biomarkers for progression of MS to T2DM by using modern technologies of systems biology (transcriptomics, metabolomics) of carefully conducted dietary interventions involving subjects with MS. The data will be analysed by using bioinformatics. We reflect these new data to well-known risk factors for T2DM and CVD, e.g. insulin sensitivity, insulin secretion, serum lipids and inflammatory factors among others. In addition to interventions conducted earlier, a new intervention with a whole grain-berry-fish diet and a control diet with refined foods will be performed. The aim is to increase our understanding on the synergistic effects of these foods, because our previous interventions have shown that these individual foods have beneficial effects on glucose and lipid metabolism. On the contrary, diets with refined foods may be harmful in long-term due to its high insulin response, which may through chronic stress lead to both insulin resistance and beta-cell damage.

The significance of this project is to increase understanding of the pathophysiology of MS, T2DM and CVD in physiological, cellular and genetic systems, which may lead to more effective and individualised strategies for treatment and prevention, and better identification of high-risk individuals responsive to specific dietary modifications. Increasing knowledge of dietary factors involved in the progression of MS to T2DM and CVD offers new opportunities to individually tailored diets in the management and prevention of these disorders. The results will also be beneficial for the food industry in developing new functional foods. These results and actions may help delay or even stop the epidemic of MS and T2DM and their negative effect on public health currently seen in Finland and worldwide.

**Key words:**  Systems biology, Nutrigenomics, Gene expression profiles, Metabolomics, Fat tissue, Peripheral mononuclear cells, Gene clusters, Personal diets, Metabolic syndrome, Type 2 Diabetes, Atherosclerosis


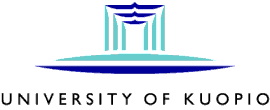

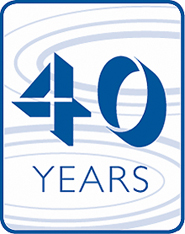


Study plan

**Systems biology approach to understand dietary modulation of gene expression and metabolic pathways in subjects with abnormal glucose metabolism (Sysdimet)**

**Applicant:**

Matti Uusitupa, M.D., Dr. Med. Sci, Professor

**Research group:**

*From the Department of Clinical Nutrition and Food and Health Research Centre, University of Kuopio, Kuopio, Finland (UKU):*

Ursula Schwab, PhD, Docent, Marjukka Kolehmainen, PhD, Leena Pulkkinen, PhD, Docent, David Laaksonen, MD, PhD, MPH, Docent, Kaisa Poutanen, DSc (Tech), Research Professor

**Co-applicant within consortium:**

Matej Oresic, PhD, Docent

**Research group of co-applicant:**

*From the VTT Technical Research Centre, Espoo, Finland (VTT):* Tuulikki Laakso-Seppänen, PhD, Docent

**International Collaborators:**

*From the Wageningen University and Reseach Centre, Wageningen, The Netherlands (WUR):* Sander Kersten, PhD, Professor

*From the University of Naples (UNA):* Gabriele Riccardi, MD, Professor

**2. TABLE OF CONTENTS**

**1. Abstract**

**2. Table of contents............................................................................................................ 2**

**3. Background.................................................................................................................... 2**

**4. Objectives....................................................................................................................... 5**

**5. Methods.......................................................................................................................... 7**

**6. Researchers and resources........................................................................................... 10**

**7. Results............................................................................................................................ 14**

**References**

**3. BACKGROUND**

**3.1. Background and significance of the research nationally and internationally**

The prevalence of metabolic syndrome (MS) and type 2 diabetes mellitus (T2DM) is increasing rapidly worldwide due to westernised dietary habits, obesity and sedentary lifestyle both in developed and developing countries. A recent survey carried out in Finland showed that the number of persons with T2DM can be as high as 12-15% among middle-aged people. Furthermore, 22-38% of middle-aged Finnish people have MS, and 30-40% have impaired fasting glucose (IFG) or impaired glucose tolerance (IGT) (Peltonen et al. 2006), both of which may lead to overt T2DM. MS is characterised by central obesity and it is closely associated insulin resistance, abnormalities in glucose and lipid metabolism, high blood pressure and inflammation, which in the long run increase the risk of T2DM and cardiovascular diseases (CVD) (Reaven 1988, Grundy 2006). Even slightly increased visceral fat mass may increase the risk of MS and its consequences, T2DM and CVD. Furthermore, increased truncal fat mass per se may be detrimental. Around 80% of persons with T2DM have also MS, most of them already years prior to the development of T2DM (Laaksonen et al. 2002, Laaksonen et al. 2004). Both MS and T2DM substantially increase the risk of CVD. MS increases the mortality and morbidity of CVD by 2- to 3-fold (Lakka et al. 2002, Laaksonen et al. 2004), while increased risk caused by T2DM could be as high as 4- to 8-fold (Uusitupa et al. 1993, Kuusisto et al. 1994).

Epidemiological studies have shown that diets high in fat, especially saturated fat, low in fiber, and rich in carbohydrates with a high glycemic index may increase the risk of T2DM and MS (Hu and Willett 2002), and that diets rich in whole grain foods reduce the risk of T2DM (Murtaugh et al. 2003, Montonen et al. 2003). In intervention studies it has been shown that both quality and amount of fat affect insulin sensitivity (Vessby et al. 2001, Lichtenstein and Schwab 2000). Furthermore, the basic mechanisms by which different nutrients may be involved in the pathogenesis of MS and T2DM are poorly understood. This concerns not only the amount and quality of dietary fat, but also the amount and quality of dietary carbohydrates. Interestingly, we have found that a diet with a low postprandial insulin response could improve early insulin response (30 min) in an OGTT (Laaksonen et al. 2005). In the Fungenut study (Table 1), we observed that gene expression in fat tissue was upregulated for several intercorrelated pathways related to metabolic stress (Kallio et al., unpublished observations). One of our hypotheses is that refined foods with high postprandial glucose and insulin response (high glycemic index foods) could be harmful in long-term due to daily repetition of high insulin response, which through chronic metabolic stress could ultimately lead both to insulin resistance and beta-cell damage. Genetic factors may contribute to this process.

The most characteristic abnormality in the MS is insulin resistance, which results from interactions between genetic and environmental factors, including diet and sedentary lifestyle (McCarthy 2004, Uusitupa 2005). The second basic abnormality in T2DM is a defect in insulin secretion, but its basic causes are poorly known. The current view is that in T2DM defects in both insulin action and insulin secretion are present, but their relative contribution varies individually. Impaired first-phase insulin secretion is present already in IFG and IGT, and it is a determinant for further progression to T2DM. The current view is that insulin secretion defect is more genetic (Uusitupa 2005), whereas insulin resistance is more closely related to fat mass, diet and other lifestyle factors. In the Finnish Diabetes Prevention Study (DPS), we have shown that insulin resistance measured during a frequently sampled intravenous glucose tolerance test (FSIGT) is highly modifiable by weight reduction and loss of fat mass, but in persons with IGT who do not progress to T2DM insulin secretion seems to be quite stable even for years (Uusitupa et al. 2003). It has been shown earlier that lifestyle interventions, including a diet high in rye bread, can improve acute insulin secretion (Juntunen et al. 2003; Laaksonen et al. 2005).

The discovery that fat tissue is an active endocrine organ has opened a new avenue to resolve the basic mechanisms involved in the development of insulin resistance in MS. Fat tissue secretes proinflammatory and prothrombotic factors such as tumor necrosis factor α (TNF-α), interleukin-6 (IL6) and plasminogen activator inhibitor 1 (PAI-1), but also insulin-sensitising factors such as adiponectin. Obesity, especially abdominal obesity, has been recognised as a low-grade inflammatory state, providing a common ground for the development of defects in glucose and lipid metabolism seen in MS and accumulating in T2DM and CVD (Yudkin 2003).

Today, we have firm evidence from four lifestyle interventions that T2DM is preventable by lifestyle changes (Pan et al. 1997; Tuomilehto et al. 2001; Knowler et al., 2002, Ramachandran et al., 2006). In DPS and Diabetes Prevention Program (DPP), there was a 58% reduction in the risk of diabetes in the intervention groups, and two other studies showed 30-40% reductions in the risk of diabetes after lifestyle modifications. Furthermore, in DPS, those persons who were able to achieve most of the goals of lifestyle changes were at markedly lower risk than those who were unable to change their lifestyles (Tuomilehto et al. 2001). Moreover, based on unpublished data on the DPS follow-up, risk reduction lasts at least a decade. Besides weight reduction and increased physical activity, also qualitative changes in the diet, i.e. increased dietary fiber consumption and the amount and quality of dietary fat may contribute to the risk reduction (Lindström et al. 2006).

Growing evidence indicates that dietary polyphenols, natural phytochemicals in plants and plant foods, such as berries and whole grain rye, influence glucose metabolism at many levels. In a recent study carried out by MTT Agrifood Research and University of Kuopio, among the 180 Finnish food items analysed, dark berries were superior sources of polyphenols (Mattila et al., unpublished observations). Red, blue and black berries contain high levels of anthocyanins, which are currently perhaps the most interesting group of polyphenols regarding modulation of glucose and lipid metabolism. The possible mechanisms include inhibition of carbohydrate digestion (McDougall et al. 2005), stimulation of insulin secretion (Jayaprakasam et al. 2005), modulation of gene expression and secretion of adipocytokines (Tsuda et al. 2004, 2006), and regulation of genes related to lipid metabolism and signal transduction (Tsuda et al. 2005). Anthocyanins may, therefore, have implications for prevention of obesity, MS and T2DM. However, these effects have not yet been demonstrated in humans.

Long chain n-3 fatty acids, abundant in fatty fish, have beneficial effects on secondary prevention of CVD. Moreover, the American and Finnish recommendations suggest certain daily intake of n-3 fatty acids in CDV patients. However, the results on the effects of n-3 fatty acids on serum glucose metabolism are inconsistent. In some studies no significant effect on serum fasting glucose concentration has been reported (Sirtori et al. 1998, Mori et al. 1999, Finnegan et al. 2003), whereas in some studies higher concentrations have been found (Woodman et al. 2002). On the contrary, fish oil supplementation has resulted in higher postprandial insulin sensitivity (Leigh-Firbank et al. 2002).

To summarise, MS, T2DM and CVD have a genetic and environmental background. This complex metabolic disturbance of still quite poorly understood. To develop new strategies for the prevention of MS and T2DM, more detailed information on the role of genetic and diet-related factors in the pathogenesis is urgently needed. Moreover, deeper understanding of the interaction among physiological, cellular and genetic levels is needed to find nutrition related early biomarkers to help identify individuals at high risk of T2DM and CVD in much earlier stage of pathogenesis (Figure 1). This knowledge offers new possibilities for the development of functional foods with beneficial effects on glucose metabolism along with creating business opportunities for diagnostic industries aiming at personalised dietary counselling.

**3.2. Previous research of the group relating to the research topic**

At the Department of Clinical Nutrition and Food and Health Research Centre, University of Kuopio, during recent years we have focused on the interaction of genes and dietary factors in the pathogenesis of MS and T2DM. Our group has carried out a number of controlled diet interventions in order to find out the effect of different dietary modifications on glucose and lipid metabolism (Sarkkinen et al., 1998; Schwab et al., 2002; Juntunen et al., 2003; Laaksonen et al., 2005). Furthermore, we have been one of the research centres of the KANWU-study, which for the first time showed in a controlled study design that a high fat diet, even independent of the quality of fat, has a detrimental effect on insulin sensitivity (Vessby et al. 2001). In DPS, we have examined the effects of different genetic factors and lifestyles (weight reduction, physical activity and diet) on the risk of T2DM. We have identified several genes that modify the effect of lifestyle changes on the risk of T2DM. In DPS, we also found that the improvement of insulin sensitivity is strongly related to weight loss in long-term and that a diet rich in saturated fat and low in fiber increases the risk of T2DM in subjects with IGT (Tuomilehto et al. 2001, Uusitupa et al. 2003, Lindström et al. 2006). Furthermore, we have also examined prospectively the impact of certain gene polymorphisms on a diet response (Sarkkinen et al. 1998, Schwab et al. 2002).

**Biomarker of an exposure**

**Biomarker of a target function**

**Biomarker of an endpoint**

**Improved**

**insulin sensitivity, blood pressure, lipid metabolism; adipokines, inflammation markers**

**Reduced risk of diabetes mellitus**

**and cardiovascular diseases**

**Dietary modification**

**Tissue gene expression, protein activation, function and tissue metabolism**

**Insulin resistance**

**Metabolic**

**syndrome**

**Abdominal obesity**

**Figure 1.** Characterization of biomarkers in the different stages of the development of metabolic syndrome.

At VTT Technical Research Centre, lipidomics has been applied extensively in studies of lipotoxicity induced insulin resistance, characterising several animal models: PPARγ2 KO (Medina-Gomez, 2005), PPARγ2KOX ob/ob (under review, Cell Metabolism), and PGC1β KO (in preparation) in collaboration with Professor Antonio Vidal-Puig, University of Cambridge. In clinical trial setting, there has been collaboration with the Type 1 Diabetes Prediction and Prevention Study (since 2004) as well as the Finnish Twin Cohort (since 2006), utilising advanced metabolomics and bioinformatics.

Currently our research at the University of Kuopio is focused on the effects of different dietary modifications on gene expression and metabolite profiles (Table 1). We have conducted two long-term dietary interventions, in which we aimed to find out to what extent weight reduction (GENOBIN) or carbohydrate modification (FUNGENUT) may modulate expression of genes in regulatory pathways associated to glucose and lipid metabolism and inflammation in subcutaneous abdominal fat tissue and peripheral mononuclear cells (PPMC). In addition, our on-going projects include two interventions, BERRY- and FISH-interventions. In the BERRY study, we examine the effects of diets rich in berries with different polyphenol profiles on glucose and lipid metabolism, and gene expression profile in PPMC. In the FISH-study our main aim is to examine the effects of fatty fish on gene expression profiles of PPMC in CVD patients.

***Five key publications***

1. Tuomilehto J, Lindström J, Eriksson JG, Valle TT, Hämäläinen H, Ilanne-Parikka P, Keinänen-Kiukaanniemi S, Laakso M, Louheranta A, Rastas M, Salminen V, Uusitupa M; Finnish Diabetes Prevention Study Group. Prevention of type 2 diabetes mellitus by changes in lifestyle among subjects with impaired glucose tolerance. N Engl J Med. 2001;344:1343-1350.

2. Laaksonen DE, Toppinen LK, Juntunen KS, Autio K, Liukkonen KH, Poutanen KS, Niskanen L, Mykkänen HM. Dietary carbohydrate modification enhances insulin secretion in persons with the metabolic syndrome. Am J Clin Nutr 2005;82:1218-1227.

3. Vessby B, Uusitupa M, Hermansen K, Riccardi G, Rivellese AA, Tapsell LC, Nalsen C, Berglund L, Louheranta A, Rasmussen BM, Calvert GD, Maffetone A, Pedersen E, Gustafsson IB, Storlien LH; KANWU Study. Substituting dietary saturated for monounsaturated fat impairs insulin sensitivity in healthy men and women: The KANWU Study. Diabetologia. 2001;44:312-319.

4. Kolehmainen M, Uusitupa MIJ, Alhava E, Laakso M, Vidal H. Effect of the Pro12Ala polymorphism in the peroxisome proliferator-activated receptor γ2 gene in the regulation of PPARγ target genes expression in adipose tissues of massively obese subjects. J Clin Endocrinol Metab, 2003;88:1717-1722.

5. Medina-Gomez G, Virtue S, Lelliott C, Boiani R, Campbell M, Christodoulides C, Perrin C, Jimenez-Linan M, Blount M, Dixon J, Zhan D, Thresher RR, Aparicio S, Carlton M, Colledge WH, Kettunen MI, Seppänen-Laakso T, Sethi JK, O’Rahilly S, Brindle K, Cinti S, Oresic M, Burcelin R and Vidal-Puig A. The link between nutritional status and insulin sensitivity is dependent on the adipocyte-specific PPAR gamma 2 isoform. Diabetes 2005;54:1706-1716.

Table 1. Dietary modification studies carried out or going on at the Department of Clinical Nutrition and Food and Health Research Centre, University of Kuopio.

| **Study/Principal investigator** | **Main aim** | **Subjects** | **Intervention** | **Main outcomes** | **Current status** |
| --- | --- | --- | --- | --- | --- |
| **Genobin**/ Uusitupa et al. | Effect of life style interventions on global gene expression in fat tissue | 75 subjects with IFG or IGT and other features of MS | Parallel study:   1. Weight reduction 2. Physical activity 3. Control   Duration 34 wks | Gene expression in fat tissue and PPMC, glucose metabolism, FSIGT | Data analysis and reporting, Salopuro et al. submitted |
| **Fungenut**/ Poutanen et al. | Effect of dietary carbohydrate modification on global gene expression in fat tissue | 47 subjects with IFG or IGT and other features of MS | Parallel study:   1. Wheat+oat+ potato 2. Rye+pasta   Duration 12 wks | Gene expression in fat tissue, glucose metabolism,  OGTT | Data analysis and reporting, Kallio et al. manuscript |
| **Berry** / Törrönen et al. | Effects of diets rich in berries on glucose and lipid metabolism and inflammation | 60 subjects with IFG or features of MS | Parallel study:  1. Strawberry, cloudberry, raspberry  2. Bilberry  3. Control  Duration 8 wks | Glucose and lipid metabolism, FSIGT, gene expression in PPMC | Intervention will be completed by July 2006 |
| **Fish** /  Erkkilä et al. | Effect of fish in secondary prevention of CVD patients | 45 subjects with AMI or AP | Parallel study:   1. Fatty fish 2. Lean fish 3. Control   Duration 8 wks | Inflammation, gene expression in PPMC | Intervention will be completed by the end of 2006 |

IFG=impaired fasting glucose, IGT=impaired glucose tolerance, MS=metabolic syndrome, PPMC=peripheral mononuclear cells, OGTT=oral glucose tolerance test, FSIGT=frequently sampled intravenous glucose tolerance test, CVD=cardiovascular diseases, AMI=acute myocardial infarction, AP=angina pectoris

**4. OBJECTIVES**

**4.1. Objective and hypothesis**

The main goal of this project is to find nutrition related early biomarkers for progression of MS to T2DM by using modern technologies of systems biology (transcriptomics, metabolomics) of carefully conducted dietary interventions involving subjects with the features of MS. Moreover, we aim to better identify high-risk individuals who are sensitive to specific dietary interventions to develop more effective and individualised treatment and prevention strategies by dietary means for MS, T2DM and CVD.

In this project we are focusing on the effects of dietary carbohydrates, whole grains, fish (n-3 fatty acids) and berries rich in polyphenols to study the possible synergistic effects of these dietary components. We also aim to identify new genes, gene clusters and biological pathways, which are related to the risk of MS and further, T2DM. The associations of these new candidate genes, gene clusters and metabolites with the risk of MS, T2DM and CVD will be confirmed in other relevant study populations, such as DPS.

***The detailed objectives are:***

1. To examine the effects of dietary modifications at the levels of molecule (gene expression, protein function), tissue (microdialysis *in vivo*), metabolome (lipidomics) and whole body physiology (biochemistry, anthropometry) (Figure 2).

2. To examine the effects of dietary modifications on gene expression in subcutaneous abdominal fat tissue and in PPMC to increase understanding how dietary factors regulate individual genes and gene clusters related to glucose and lipid metabolism and inflammation.

3. To explore biomarkers for early detection of the disease pathogenesis (Figure 1) using modern bioinformatics.

4. To examine the association of new interesting genes identified by microarray gene expression studies to the risk of T2DM.

5. To characterise specific functions and mechanisms for genes responding to dietary modification by using *in vitro* cell culture models.

6. To apply bioinformatics in evaluating dietary effects of global gene expression and lipidomics and to combine these data with clinical measurements.

Our hypothesis is that dietary modifications induce beneficial changes at levels of molecule, tissue, metabolome and whole body physiology, and by combining the results from these levels it is possible to find early biomarkers for disease pathogenesis and identify individuals sensitive to diet modification.

Molecular level:

DNA, mRNA, protein/UKU

Tissue level

*in vivo*:

microdialysis/ UKU

Metabolomics/

VTT

Whole body:

glucose, lipid and adipose tissue metabolism/ UKU

**Reduced risk of diabetes mellitus**

**and cardiovascular diseases**

**Data collection**

**completed:**

**Genobin/UKU**

**Fungenut/UKU**

**Ongoing:**

**Fish/UKU**

**Berry/UKU**

**Collaboration**

**/UNA**

**Intervention**

**to be carried out**

**2007-2008/UKU**

**Blood, fat tissue and peripheral mononuclear cells**

**Bioinformatics/ WUR/ VTT/ UKU**

- sensitive genotype

- gene expression

- gene function

- regulation of lipolysis

- glucose uptake

- changes in lipid profile

- insulin sensitivity

and secretion

- glucose concentration

- lipid and lipoprotein

concentrations

- blood pressure

- adipokines

- inflammatory markers

**Figure 2.** Study flow: interventions, study collaborators, samples collected and outcome measures. UKU=University of Kuopio, UNA=University of Naples, VTT=VTT Technical Research Centre, WUR=Wageningen University and Reasearch Centre.

**4.2. Compatibility with the ELVIRA-program and expected impact on society**

The project addresses mainly the research theme “Nutrition, genetic factors and metabolism”, and to some extent also the theme “Food processing and health”. It deals with a major diet-linked public health problem in Finland and globally, and builds a new international network to support the development of the use of new technologies and approaches to address it. It includes both student and post-doctoral education, and has a program for international exchange. The consortium has ecellent potential to be one of the international leaders in the chosen research field.

The significance of this study is to increase understanding of the regulatory mechanisms of MS, T2DM and CVD at the level of physiological, cellular and genetic systems that may lead to more effective and individualised new treatment and prevention strategies and identification of high-risk individuals especially responsive to specific dietary modifications. Increasing knowledge of dietary factors involved in the progression of MS to T2DM and CVD offers new opportunities to individually tailored diets in the management and prevention of these disorders. It also offers new possibilities for development of functional foods with beneficial effects on glucose metabolism, and creates business opportunities for diagnostic industries aiming at personalised dietary counselling. The resulting knowledge may help delay or even stop the epidemic of MS and T2DM and their negative effect on public health currently seen in Finland and in most affluent societies worldwide. This project produces high quality and innovative information on the health effects of different dietary means in promoting health and prevention of chronic diseases. Dietary means have no side effects and have in many cases also shown to be cost efficient. Their potential in prevention and treatment of chronic diseases is, however, clearly under-exploited.

**5. METHODS**

**5.1. Interventions**

***Previous studies.*** Above mentioned GENOBIN and FUNGENUT studies are in the phase of data handling. Table 1 summarises the study populations of those interventions carried out in years 2003-2006. The preliminary results of the GENOBIN and FUNGENUT studies show diet induced changes in glucose metabolism and fat tissue gene expression. Specifically, weight reduction and low insulin response diet resulted in a global downregulation in gene expression (Salopuro et al. submitted, Kallio et al. unpublished), e.g. the tenomodulin (TNMD) gene. The expression levels of the TNMD correlated inversely with insulin sensitivity. Subsequently, the association of this gene with conversion of IGT to T2DM was then analysed in the DPS. We found a strong association of three TNMD SNPs with conversion of IGT to T2DM in men (Pulkkinen et al. submitted).

Metabolites as physiological endpoints may be particularly suitable for characterisation of complex interventions such as in nutrition. We have performed initial serum lipid profiling in the GENOBIN study, which lead to identification of changes of lipid profiles specific to intervention group. Preliminary analyses of serum lipidomics data from GENOBIN study reveal changes in lipid profiles as a result of intervention which can be attributed to redistribution of specific lipid classes.

***Ongoing studies.*** BERRY-study: Subjects 25-60 years of age with features of metabolic syndrome were randomised according to age, BMI and fasting plasma glucose concentration into one of the three study groups: Group A with 300-400 g of strawberry, cloudberry and raspberry/day, Group B with 300-400 g of bilberry/day and Group C (control group) with <1 dl of berries/day.

FISH-study: Subjects under 70 years of age who have had an acute myocardial infarction or acute coronary event requiring hospitalisation during the past 3-24 months have been identified from the hospital discharge lists at the Kuopio University Hospital. The subjects are randomly assigned to one of the three groups: control, white (lean) fish and fatty fish. During all the diet phases, the intake of nutrients other than n-3 fatty acids follows the general recommendations for CVD patients.

***Diet intervention to be conducted during the present project.*** This new dietary modification study aims to combine the beneficial effects of diet components found in previous diet interventions. Here we will investigate the possible synergistic effects of whole grain, fish and berries on glucose and lipid metabolism, and on gene expression in PPMC and fat tissue. Furthermore, microdialysis will be performed to examine the effects at fat tissue level *in vivo*.

**Group A: Whole grain + berries + fish**

🡱

🡱

🡱

🡱

**0 wk**

**4 wk**

**8 wk**

**12 wk**

**Health Grain: Whole grain**

**Group B: Control diet, refined foods**

**Intervention/UKU**

**HealthGrain/UKU&UNA**

Anthropometry

Blood samples:

-biochemistry

-lipidomics

-genotyping

Gene expression:

-PPMC

-Fat tissue biopsy

Microdialysis

OGTT, FSIGT

4 day food record

Anthropometry

Blood samples:

-biochemistry

-lipidomics

Gene expression:

-PPMC

-Fat tissue biopsy

Microdialysis

OGTT, FSIGT

4 day food record

Anthropometry

Blood samples:

-biochemistry

4 day food record

Anthropometry

Blood samples:

-biochemistry

4 day food record

**Figure 3.** Study design in the diet intervention. UKU=University of Kuopio, UNA=university of Naples, PPMC= peripheral mononuclear cells, OGTT=oral glucose tolerance test, FSIGT=frequently sampled glucose tolerance test.

The diet intervention will be performed according to a parallel study design (Figure 3). Sixty subjects aged 40-65 years will be recruited and randomly assigned to two groups (A, B) matched by age, gender, BMI and 2-h glucose concentration in an oral glucose tolerance test (OGTT). The diet intervention lasts for 12 weeks. In group A, the subjects will follow a diet based on the general nutrition recommendations (American Heart Association, Finnish Heart Association). They will also be instructed to consume whole grain products 25 % of energy intake and berries 300 g/day. In addition, they will have fish meals 4 times per week. In group B, the subjects will follow refined food diet with restricted intake of berries, fish and whole grain products. The subjects will be instructed by a clinical nutritionist. The third arm (Figure 3) will be performed in the consortium of Health Grain (EU, Prof. Riccardi). The data will be pooled with the results from the present proposal to examine and compare the effects of whole grain and refined foods diets on the gene and lipid profiles. The subjects keep 4-day food records three times during the study: before the intervention and during the weeks 3, 7 and 11. Food records will be analysed by the Nutrica® dietary analysis software.

***Biochemical measurements*.** The FSIGT will be performed according the Minimal Model method (Bergman 1989). A 2h OGTT will be performed as well. Blood samples will be analysed by the methodology currently in use at the Department of Clinical Nutrition and Clinical Research Unit at the University of Kuopio and the Clinical Chemistry Laboratory at the Kuopio University Hospital.

***Peripheral mononuclear cells*** are isolated using BD vacutainer CPT, Cell Preparation Tubes according to instructions of the manufacturer (Beckton Dickinson). The isolated cells are suspended to lysis buffer of RNA extraction kit and stored at -80ºC until to be analysed.

***Fat tissue samples*** for the gene expression analysis will be taken under local anesthesia from subcutaneous abdominal fat tissue by needle biopsy. The site of the biopsy is the midpoint of the umbilicus and suprailiaca, both on the left and the right side of the body. Samples will be immersed immediately into liquid nitrogen and stored in -80ºC until analysed.

***Genetic association studies/Genotyping.*** The genes responding to diets based on gene expression studies are considered as candidate genes for genetic association studies (Figure 4). The most informative set of single nucleotide polymorphisms (SNPs) from each haploblock of each gene are selected from HapMap-database (hapmap.org). The association of the SNPs with conversion of IGT to T2DM is the main interest, but biochemical markers and anthropometric measurements relevant for detecting risk factors for the T2DM, such as the status of glucose intolerance and insulin resistance and central obesity will also be analysed. The SNP analysis will be done with Taqman chemistry based assays available in Applied Biosystems. Allelic discrimination will be analysed with ABI 7000 equipment.

**INTERVENTIONS**

**Gene expression, Lipidomics, Bioinformatics**

**Candidate Genes**

**Figure 4.** The flow for functional and genetic studies.

***Gene expression****.* For mRNA expression studies total RNA will be extracted using commercially available kits (RNeasy Minikit® or Midikit®, Qiagen; Trizol®, Invitrogen) from fat tissue and PPMC samples. Microarray analysis will be done from total RNA of 10 individuals/study group using HG-U133 Plus 2.0 GeneChip -microarrays (Affymetrix Inc., Santa Clara, CA, USA). Hybridisations and scannings will be performed in Affymetrix facility. Quantitative PCR for selected genes will be performed with ABI 7500 (Applied Biosystems). Assays with Taqman chemistry will be used.

***Protein analysis.*** Fat tissue samples will be stored at -80ºC for protein analyses. Proteins that correspond to genes which display up- or down-regulation by microarray analysis and that are confirmed by real time PCR, are selected for protein analysis by using specific antibodies in order to study activation/inactivation of relevant signaling proteins and to examine correlation between RNA and protein expression.

***In vitro studies.*** The *in vitro* studies aim to explore the specific mechanisms, how the genes responding to dietary modifications modulate glucose metabolism and insulin sensitivity at molecular level. The candidate genes for these studies are obtained from Affymetrix data of the interventions (Figure 4). We will use primary human adipocytes (Cambrex, Promocell) or a murine adipocyte cell line 3T3-L1 as model systems. The functional studies include model systems that allow either over-expression of the candidate under a strong promoter (CMV) or suppression of its expression by using RNA interference (RNAi) technology. The effects of expression levels on specific features of adipocytes will be studied by a) proliferation b) differentiation, and c) apoptosis assays. Furthermore, the effects of expression levels on insulin sensitivity will be studied by d) insulin-mediated glucose uptake assay, e) studying expression and posttranslational modifications (phosphorylation, protein processing) of the target molecules of insulin pathway (i.e. insulin receptor substrate 1 (IRS1) and glucose transporter 4 (GLUT4), and f) lipid metabolism including liponeogenesis and lipolysis.

***In vivo tissue metabolism.*** Fat tissue metabolism *in vivo* will be measured using microdialysis technique (Lafontan and Arner 1996). In these experiments, glucose metabolism and lipolytic activity of subcutaneous abdominal fat tissue *in situ* will be determined. This measurement will be done in a sub-sample of subjects (10 individuals/study group) participating in the dietary intervention. Needle-like catheters with semipermeable membrane will be inserted in subcutaneous fat tissue. Small concentrations of agents will be perfused into the tissue for studying stimulation or inhibition of lipolysis, and glucose metabolism *in vivo*. Dialysate glycerol, glucose, lactate and pyruvate will be determined. Blood flow will be determined using ethanol dilution method throughout the study.

***Metabolomics.*** The analytical platforms for metabolomics can be roughly divided into global screening and targeted platforms. The screening platforms include 1) lipidomics platform, covering 500+ sterol-esters, acylglycerols, phospholipids, and sphingolipids within a single sample run, and 2) screening platforms for water soluble metabolites using Ultra Performance Liquid Chromatography (UPLC/MS). Depending on specific needs, we have been also developing targeted platforms for specific groups of compounds such as eicosanoids and free fatty acids, sterols, sphingolipids, nucleotides, citric acid cycle metabolites, sugar phosphates, and others. For biomarker discovery and characterisation of interventions it is common to start with at least one of the screening platforms, followed by more targeted approaches if necessary.

***Bioinformatics.*** Analysis of microarray data will be done in collaboration with the Nutrition, Metabolism and Genomics group at Wageningen University and Research Centre (Prof. Kersten). Quality control of microarray data will be performed by making M vs. A plots of biological replicates. The optimal normalisation strategy will be determined using GC-RMA method. Appropriate statistical analysis to determine differentially expressed genes will be performed. In addition to p-values, q-values (false discovery rate) will be determined. Thereafter, cluster and/or pathway analysis will be carried out (GenMapp, Ingenuity etc.).

Both univariate and multivariate data analyses will be applied for metabolomics data analysis. Regression and correlation network analyses will be utilised to combine these data with the clinical data. Pathway analysis methods (Curtis et al, 2005) will be utilised for analysis of gene expression data, and such information will be correlated with serum metabolic patterns obtained.

**5.2. Schedule of the project**

|  | **2007** | **2008** | **2009** | **2010** |
| --- | --- | --- | --- | --- |
| Screening of subjects for the intervention | x |  |  |  |
| Diet intervention |  | x | x |  |
| Whole body level measurements |  | x | x |  |
| Analysis of microdialysis |  | x | x |  |
| Genotyping and gene expression | x | x | x | x |
| Lipidomics | x | x | x | x |
| Bioinformatics | x | x | x | x |
| Biomarkers | x | x | x | x |
| Reporting the results | x | x | x | x |

**5.3. Ethical and data protection issues**

The previously conducted interventions have been performed according to ethical principles and the study plans have been approved by the Ethics Committee of the District Hospital Region of Northern Savo and National Institute of Public Health. The study plan of the new intervention will be sent to the Ethics Committee of the District Hospital Region of Northern Savo for approval. Regarding subject information security, all current ethical instructions have been and will be strictly followed. The approvals of the Ethics Committees are DPS 6.3.1992/National Institute of Public Health /Helsinki, and Genobin 152/2002, Fungenut 54/2003, Fish 110/2005, and Berry 124/2005, Kuopio.

**6. RESEARCHERS AND RESOURCES**

**6.1. Composition of consortium**

This study project will be carried out at the Department of Clinical Nutrition and Food and Health Research Centre, University of Kuopio in collaboration with VTT Technical Research Centre, Wageningen University and Research Centre and University of Naples.

***University of Kuopio (UKU). Matti Uusitupa***, M.D., Professor, applicant, leader of the project: responsible for the expertise in pathophysiology of MS and T2DM and connections to the Center of Excellence for Research in Cardiovascular Diseases and Type 2 Diabetes of the Academy of Finland. Professor Uusitupa is also the principal investigator in the gene/lifestyle interaction studies in the Finnish DPS. ***Ursula Schwab***, PhD, Docent, authorised nutritionist: responsible for the intervention and clinical data. ***Marjukka Kolehmainen***, PhD, authorised nutritionist: responsible for gene expression studies and fat tissue measurements *in vivo*. ***David Laaksonen***, MD, PhD, MPH, Docent: responsible for clinical examinations. ***Leena Pulkkinen***, PhD, Docent, Research manager: responsible for molecular genetics. ***Kaisa Poutanen***, DSc (Tech), Research Professor: food characteristics and technology, responsible for the connections to the HEALTHGRAIN program

The background supporting group is formed by Hannu Mykkänen, PhD, Professor, Arja Erkkilä, PhD, Docent, and Riitta Törrönen, PhD, Docent, with long term scientific expertise on their specific fields.

Members of the research team will be mutually responsible for mentoring doctoral students in this project and participate in the analysis and interpretation of the data collected and writing the papers.

***VTT, Technical Research Centre (VTT). Matej Oresic***, PhD, Docent, co-applicant: responsible for metabolomics, bioinformatics. ***Tuulikki Seppänen-Laakso***, PhD, Docent: responsible for lipid analytics.

***International collaborators.*** Professor ***Sander Kersten*** from the ***Wageningen University and Research Centre (WUR)***, Nutrition, Metabolism and Genomics group is responsible for the analysis of microarray data. The Nutrition, Metabolism and Genomics group at Wageningen University are a key group within the EU NoE NUGO  (European Nutrigenomics organisation). They also direct a large national nutrigenomics program (Nutrigenomics consortium) that focuses on metabolic syndrome.  The group specialises in transcriptomics and runs their own Affymetrix platform. In this project the group  will participate in the bioinformatics analysis of the data, and has also agreed to supervise visiting scientists of the consortium.

Professor ***Gabriele Riccardi***, Professor of Endocrinology and Metabolic Diseases, ***University of Naples (UNA)***. He is chief of the Diabetes Clinic and of the Metabolic Ward at the University Hospital and Chairman of the University degree on Dietetics. He has deep expertise in the field of diabetes research. He has been a chair of the International Task Group on obesity, insulin sensitivity and diabetes mellitus of the EU PASSCLAIM project. In this project, Prof. Riccardi will be a partner of the two-centre whole grain intervention and participate in analysis of results.

***Added value of the consortium.*** At the Department of Clinical Nutrition and the Food and Health Research Centre, University of Kuopio has a long term expertise and excellent facilities in conducting diet intervention studies (International evaluation on food sciences and related research in Finland 2002-2004, Academy of Finland 02/2006). Due to the most recent development in the technologies of nutrigenomics special expertise is needed to process and interpret the results. The expertise of VTT and WUR will be essential in data processing and interpretation. Our previous findings show how powerful these new technologies can be when studying gene expression and metabolite profiling in defining the impact of dietary and lifestyle interventions on the regulatory pathways of biological systems. These approaches can be expected to provide deeper insight into the mechanisms behind disturbed glucose and lipid metabolism and inflammation in MS, T2DM and CVD, and improved targeting of high risk individuals particularly responsive to specific dietary interventions. The work carried out by Professor Gabriele Riccardi within the consortium is to ascertain the effects in individuals with different genetic and life-style background. Interactions of diet, lifestyle factors and genetic background will be determined, and the feasibility of the whole grain concept in advancing health will be tested in conditions representing the northern and southern European dietary and lifestyle habits.

**6.2. Plans for collaboration**

Consumers' attitudes towards personalised diets and social acceptance of them are very important. For the beneficial effects of dietary modifications to be taken into practice, it is essential that consumers feel safe and comfortable towards tailored diets. Thus, within the present proposal these aspects will be taken into account by collaboration with the consortium of "Consumers and healthy eating: risk, individualisation and illness prevention (COHERI)" presented to Academy of Finland as an ELVIRA-proposal and coordinated by Johanna Mäkelä, D.Soc.Sc., Head of Research, more specifically within the sub-study of the proposal, "Nutrigenomics and the individualisation of eating (NUTGEN)" by senior researcher Mari Niva, in the National Consumer Research Centre.

UKU and Food and Health Research Centre is involved (Leena Pulkkinen et al.) in the ELVIRA-proposal consortium "Food Phytochemicals" coordinated by Sari Mäkelä from University of Turku, which complements the current proposal by assessing the bioavailability, metabolism and mechanisms of phytochemicals in cellular, animal and short interventions studies.

**6.3. Other ongoing projects in which the researchers are involved and their funding**

Professor Uusitupa's group has been involved in studies on the genetic background of T2DM and diet-gene and gene-gene interactions for years. The basic money for these studies is from the Center of Excellence for Research in Cardiovascular Diseases and Type 2 Diabetes of the Academy of Finland (339 600 €, 2003-2006). Genobin study has been financially supported by Sigrid Juselius Foundation (250 000 €, 2004-2009) and EVO funding from the Kuopio University Hospital (211 500 €, 2003-2006). Fungenut study has been supported by Tekes, Finland (225 000 €, 2003-2005) and by Juho Vainio Foundation (10 000 €, 2004). Berry study is funded by Tekes (30 000 €, 2006, coordinated by VTT/Berrydrug-project) and by Juho Vainio Foundation (10 000 €, 2006). Fish study is funded by Juho Vainio Foundation (10 000 €, 2005), the Finnish Cultural Foundation (10 000 €, 2005), The Finnish Cultural Foundation (Northern Savo Fund: 10 000 €, 2005) and Yrjo Jahnsson Foundation (8 000 €, 2004). Marjukka Kolehmainen has post doctoral funding from the Academy of Finland (186 160 € for May 2005 to April 2008). Other public foundations have supported the research activity by 90 000 € during the past years.

Within the EU FP6 IP project "HEALTHGRAIN (2005-2010)" the breeding, processing, and metabolism of mainly wheat but also to a lesser extent rye will be studied to better exploit the potential of whole grain for prevention of MS. Healthgrain will form an interactive collaboration environment for the present proposal. The Kuopio research group (Prof. Hannu Mykkänen) is responsible for the work package on dietary interventions, and Kaisa Poutanen is the coordinator of the project.

The EU FP6 IP project “HEPADIP (2005-2010)” aims to address the role of adipose tissue and the liver, and the interaction between them, in the development of the disturbances of lipid metabolism, insulin signalling, and glucose homeostasis in MS in order to identify, validate and develop novel targets for diagnosis, characterisation, prevention and treatment of the syndrome. The VTT Quantitative Biology and Bioinformatics group (Oresic) is responsible for lipidomics and related bioinformatics in the HEPADIP project.

**6.4. Research training, arrangements for supervision**

The Department of Clinical Nutrition at the University of Kuopio has provided clinical nutrition curriculum since 1984 leading to MSc and PhD degrees in Nutrition or Clinical Nutrition. The Department has the only chair in clinical nutrition among the Universities of Finland. The research group of the present proposal are active participants in the Finnish Graduate School in Applied Bioscience - Bioengineering, Food & Nutrition, Environment (ABS Graduate School) coordinated by the University of Helsinki. Professor Poutanen and Professor Mykkänen are in the management board of the ABS School. Researcher training is currently organised through post-graduate courses, monthly seminars and via laboratory meetings within the individual research groups.

***PhD students of the present proposal.*** Tiina Lappalainen M.Sc. is being supervised by Prof. Helena Gylling, Ursula Schwab and Marjukka Kolehmainen. Anna-Maija Tolppanen M.Sc. is being supervised by Matti Uusitupa, Leena Pulkkinen and Marjukka Kolehmainen. Two new PhD students will be recruited and will be supervised by the senior scientists. One of them will be trained in collaboration with VTT Quantitative Biology and Bioinformatics group. In addition, the results of this project will form part of the PhD theses of Titta Salopuro, M.Sc. (supervis. MU, LP, MK), Niina Siitonen, M.Sc. (MU, LP) and Petteri Kallio, M.Sc. (Kaisa Poutanen, LP, MU).

***Post Doctoral positions of the present proposal.*** Marjukka Kolehmainen, PhD, will complete her post doctoral period during the present proposal. The continuation of the funding after the period has been applied in this proposal. Vanessa Derenji Ferreira de Mello is doing her post doctoral period at the Department of Clinical Nutrition in the Genobin and Fish studies. She has completed her PhD studies at the Department of Internal Medicine, Medical School, Federal University of Rio Grande do Sul (UFRGS), Porto Alegre, Brasil.

**6.5. Planned mobility of researchers**

As a part of the researcher training, both in the stage of PhD studies and post doctoral period, training exchange is planned among VTT, WUR and UKU. Lipidomics and bioinformatics will be part of the PhD thesis of a PhD student recruited for the present study. His/her training will be planned to cover clinical part of the new intervention to be conducted during the present proposal as well as the lipidomic analyses in laboratory and the bioinformatic part in collaboration between VTT and UKU. WUR has special expertise in gene expression profiling and data handling.

**6.6. Research environment**

This study will be carried out at the *Department of Clinical Nutrition* and *Food and Health Research Centre (ETTK)*, University of Kuopio. These units will provide the necessary facilities such as laboratory space, equipment and personal computers. There are excellent facilities to carry out intervention trials as well as to analyse both biochemical and food record data. There are also modern facilities to perform gene polymorphism and expression analyses (core facilities of the Mediteknia building). Part of the analyses (Affymetrix Inc.) will be carried out at the A.I. Virtanen Institute, University of Kuopio, which has the Affymetrix equipment and other necessary facilities available for all research groups at the University.

*VTT Technical Research Centre* provides resources and infrastructure for metabolomics including one QTof based LC/MS system (QTof Premier with UPLC Acquity chromatography), one triple quadrupole based LC/MS system, and one GC/MS system. In May 2006 infrastructure is being expanded with a new LC/MS system (linear ion trap MS), GCxGC-Tof system, and Advion Nanomate nanoelectrospray chip system. The infrastructure is complemented by in house developed softwares for data processing (Katajamaa and Oresic, 2005) and a sophisticated chemo-/bioinformatics database system for data storage and interpretation (Gopalacharyulu et al, 2005).

**6.7. Financial plan for the project**

Personnel to be funded by the project

| Researchers  Technicians  Laboratory nurses  PhD students  **Total costs** | 2.5 person years  1.5 py + 4 person months  1.5 person years  8 person years | 115 000 €  64 200 € *  49 500 €  224 000 € *  **452 700 €**  **321 500 €/UKU** |
| --- | --- | --- |

Materials and equipment

| Analyses of blood samples  Microdialysis  Adipose tissue biopsies  RNA and DNA isolations  Genotyping  Quantitative PCR  Lipidomics  Microarray  Researcher exchange  Congresses  **Total costs** |  | 50 000 €  35 000 €  3 000 €  7 000 €  40 000 €  40 000 €  20 000 €*  108 000 €  10 000 €  7 000 €    **320 000 €**  **300 000 €/UKU** |
| --- | --- | --- |

**Total funding applied from the Academy of Finland within consortium 772 700 €**

**Total funding applied for UKU 621 500 €**

***Total funding applied for VTT 151 200 €**

**Persons to be hired at UKU:**

Tiina Lappalainen, M.Sc., authorised nutritionist 28000 € (1 py)

Anna-Maija Tolppanen, M.Sc., 28 000 € (1 py)

NN, M.Sc., 56 000 € (2 py)

Marjukka Kolehmainen, PhD, 115 000 € (2.5 py)

Laboratory nurse, 49500 € (1.5 py)

Technician, 45 000 € (1.5 py)

***Co-applicant Matej Oresic will apply own funding within this consortium including:**

Technician 19 200 € (4 person months)

PhD student 112 000 € (4 py)

Consumables for lipidomics analysis 20 000 €

Total 151 200 €

**6.8. Justification for application for funding**

The present proposal provides new, innovative knowledge concerning the effects of dietary modification on the different levels of molecule, tissue, metabolome and whole body physiology applying modern technologies. These technologies are high in expenses and thus, not fundable from private foundations. Furthermore, these new technologies are laborous and the demand of personnel is higher than in conventionally performed and analysed studies. Funding from the Academy of Finland will play a crucial role in the present proposal, where modern technologies will be applied efficiently for the use of nutritional and food sciences. These technologies are regarded necessary for the development of the research area.

**7. RESULTS**

**7.1. Expected results and their scientific significance**

Our results will provide new data on the impact of dietary modifications and dietary ingredients on the molecular, cellular, metabolic and whole body level. We will apply bioinformatics technologies and techniques that make it possible to combine data from different levels (gene expression, proteomics, metabolomics, whole body physiology). Furthermore, we will identify new genes and gene clusters to be tested in different clinical population based materials, e.g. DPS. We will deepen the knowledge of the gene-diet and gene-gene interactions in the development of MS, T2DM and CVD. This systems biology approach is very powerful. Our collaboration and data collection from different interventions to increase sample size makes it possible to analyse even complex associations and interactions..

**7.2. Practical applicability of research results**

The main aim of this project is to provide new information of more tailored diets in the prevention of MS, T2DM and CVD. Identifying new pathways is crucial in developing new agents for the prevention/treatment of MS, T2DM and CVD. This line of research lasts for years, but it also has major commercial potential. Based on the preliminary results from the Genobin study, we have already started this kind of research with a new potential gene that may be involved in the development of diabetes in men. Learning about the food-induced changes will enable food industry to utilise the new data when developing healthy foods and individualised dietary services. The results may also be used in development of new diagnostic tools to identify individuals sensitive to dietary treatment.

**7.3. Publication plan and other dissemination**

This project will generate 30-40 papers published in peer-reviewed international journals of the field. Two doctoral dissertations will be prepared from the results of this project. Furthermore, at least 3 doctoral dissertation will include results from this project. We will also continue to disseminate the results and the concept of nutrigenomics to the food industry and to large public in targeted seminars and trade journals.

**REFERENCES**

Bergman RN. Toward physiological understanding of glucose tolerance. Minimal-Model approach. Diabetes 1989;38:250-256.

Curtis K, Orešič M, Vidal-Puig A. Pathways to analysis of microarray data. Trends Biotechnol 2005;8: 429-435.

Finnegan YE, Minihane AM, Leigh-Firbank EC, Kew S, Meijer GW, Muggli R, Calder PC, Williams CM. Plant- and marine-derived n-3 polyunsaturated fatty acids have differential effects on fasting and postprandial blood lipid concentrations and on the susceptibility of LDL to oxidative modification in moderately hyperlipidemic subjects. Am J Clin Nutr 2003;77:783-795.

Gopalacharyulu PV, Lindfors E, Bounsaythip C, Kivioja T, Yetukuri L, Hollmén J, Orešič M. Data integration and visualization system for enabling conceptual biology. Bioinformatics 2005;21:i177-i185.

[Grundy SM.](http://www.ncbi.nlm.nih.gov/entrez/query.fcgi?cmd=Retrieve&db=pubmed&dopt=Abstract&list_uids=16545636&query_hl=10&itool=pubmed_docsum) Metabolic syndrome: connecting and reconciling cardiovascular and diabetes worlds.
J Am Coll Cardiol 2006;47:1093-1100.

Hu FB, Willett WC Optimal diets for prevention of coronary heart disease. JAMA 2002;288:2569-2578.

[Jayaprakasam B, Vareed SK, Olson LK, Nair MG.](http://www.ncbi.nlm.nih.gov/entrez/query.fcgi?cmd=Retrieve&db=pubmed&dopt=Abstract&list_uids=15631504&query_hl=18&itool=pubmed_docsum) Insulin secretion by bioactive anthocyanins and anthocyanidins present in fruits. J Agric Food Chem 2005;53:28-31.

Juntunen KS, Laaksonen DE, Autio K, Niskanen LK, Holst JJ, Savolainen KE, Liukkonen KH, Poutanen KS, Mykkänen HM. Structural differences between rye and wheat breads but not total fiber content may explain the lower postprandial insulin response to rye bread. Am J Clin Nutr 2003;78:957-964.

Katajamaa M, Orešič M. Processing methods for differential analysis of LC/MS profile data. BMC Bioinformatics 2005;6:179(1-12).

[Knowler WC, Barrett-Connor E, Fowler SE, Hamman RF, Lachin JM, Walker EA, Nathan DM. Diabetes Prevention Program Research Group.](http://www.ncbi.nlm.nih.gov/entrez/query.fcgi?cmd=Retrieve&db=pubmed&dopt=Abstract&list_uids=11832527&query_hl=46&itool=pubmed_docsum) Reduction in the incidence of type 2 diabetes with lifestyle intervention or metformin. N Engl J Med 2002;346:393-403.

Kolehmainen M, Uusitupa MIJ, Alhava E, Laakso M, Vidal H. Effect of the Pro12Ala polymorphism in the peroxisome proliferator-activated receptor γ2 gene in the regulation of PPARγ target genes expression in adipose tissues of massively obese subjects. J Clin Endocrinol Metab 2003;88:1717-1722.

[Kuusisto J, Mykkänen L, Pyörälä K, Laakso M.](http://www.ncbi.nlm.nih.gov/entrez/query.fcgi?cmd=Retrieve&db=pubmed&dopt=Abstract&list_uids=8039603&query_hl=13&itool=pubmed_docsum) NIDDM and its metabolic control predict coronary heart disease in elderly subjects. Diabetes 1994;43:960-967.

[Laaksonen DE, Lakka HM, Niskanen LK, Kaplan GA, Salonen JT, Lakka TA.](http://www.ncbi.nlm.nih.gov/entrez/query.fcgi?cmd=Retrieve&db=pubmed&dopt=Abstract&list_uids=12446265&query_hl=11&itool=pubmed_docsum) Metabolic syndrome and development of diabetes mellitus: application and validation of recently suggested definitions of the metabolic syndrome in a prospective cohort study. Am J Epidemiol 2002;156:1070-1077.

[Laaksonen DE, Niskanen L, Lakka HM, Lakka TA, Uusitupa M.](http://www.ncbi.nlm.nih.gov/entrez/query.fcgi?cmd=Retrieve&db=pubmed&dopt=Abstract&list_uids=15478308&query_hl=11&itool=pubmed_docsum) Epidemiology and treatment of the metabolic syndrome. Ann Med 2004;36:332-346.

[Laaksonen DE, Toppinen LK, Juntunen KS, Autio K, Liukkonen KH, Poutanen KS, Niskanen L, Mykkänen HM.](http://www.ncbi.nlm.nih.gov/entrez/query.fcgi?cmd=Retrieve&db=pubmed&dopt=Abstract&list_uids=16332654&query_hl=15&itool=pubmed_docsum) Dietary carbohydrate modification enhances insulin secretion in persons with the metabolic syndrome. Am J Clin Nutr 2005;82:1218-1227.

Lafontan M, Arner P. Application of in situ microdialysis to measure metabolic and vascular responses in adpose tissue. Trends Pharmac Sci 1996;17:309-313.

[Lakka HM, Laaksonen DE, Lakka TA, Niskanen LK, Kumpusalo E, Tuomilehto J, Salonen JT.](http://www.ncbi.nlm.nih.gov/entrez/query.fcgi?cmd=Retrieve&db=pubmed&dopt=Abstract&list_uids=12460094&query_hl=11&itool=pubmed_docsum) The metabolic syndrome and total and cardiovascular disease mortality in middle-aged men. JAMA 2002;288:2709-2716.

Leigh-Firbank EC, Minihane AM, Leake DS, Wright JW, Murphy MC, Griffin BA, Williams CM. Eicosapentaenoic acid and docosahexaenoic acid from fish oils: differential associations with lipid responses. Br. J. Nutr. 2002;87:435-45.

Lichtenstein AH, Schwab US. Relationship of dietary fat to glucose metabolism. Atherosclerosis 2000;150:227-243.

Lindström J, Peltonen M, Eriksson JG, Louheranta A, Fogelholm M, Uusitupa M, Tuomilehto J. High-fibre, low-fat diet predicts long-term weight loss decreased type 2 diabetes risk: the Finnish Diabetes Prevention Study. Diabetologia 2006;49:912-920.

McCarthy MI. Progress in defining the molecular basis of type 2 diabetes mellitus through susceptibility-gene identification. Hum Mol Genet 2004;13, Review issue 1:R33-R41.

[McDougall GJ, Shpiro F, Dobson P, Smith P, Blake A, Stewart D.](http://www.ncbi.nlm.nih.gov/entrez/query.fcgi?cmd=Retrieve&db=pubmed&dopt=Abstract&list_uids=16498205&query_hl=17&itool=pubmed_docsum) Different polyphenolic components of soft fruits inhibit α-amylase and α-glucosidase. J Agric Food Chem 2005;53:2760-2766.

Medina-Gomez G, Virtue S, Lelliott C, Boiani R, Campbell M, Christodoulides C, Perrin C, Jimenez-Linan M, Blount M, Dixon J, Zhan D, Thresher RR, Aparicio S, Carlton M, Colledge WH, Kettunen MI, Seppänen-Laakso T, Sethi JK, O’ Rahilly S, Brindle K, Cinti S, Oresic M, Burcelin R, Vidal-Puig A. The link between nutritional status and insulin sensitivity is dependent on the adipocyte-specific PPAR gamma 2 isoform. Diabetes 2005;54:1706-1716.

Mori TA, Bao DQ, Burke V, Puddey IB, Watts GF, Beilin LJ. Dietary fish as a major component of a weight-loss diet: effect on serum lipids, glucose, and insulin metabolism in overweight hypertensive subjects. Am J Clin Nutr 1999;70:817-825.

Montonen J, Knekt P, Järvinen R, Aromaa A, Reunanen A. Whole-grain and fiber intake and the incidence of type 2 diabetes. Am J Clin Nutr 2003;77:622-629.

Murtaugh MA, Jacobs DR Jr, Jacob B, Steffen LM, Marquart L. Epidemiological support for the protection of whole grains against diabetes. Proc Nutr Soc 2003;62:143-149.

[Pan XR, Li GW, Hu YH, Wang JX, Yang WY, An ZX, Hu ZX, Lin J, Xiao JZ, Cao HB, Liu PA, Jiang XG, Jiang YY, Wang JP, Zheng H, Zhang H, Bennett PH, Howard BV.](http://www.ncbi.nlm.nih.gov/entrez/query.fcgi?cmd=Retrieve&db=pubmed&dopt=Abstract&list_uids=9096977&query_hl=45&itool=pubmed_docsum) Effects of diet and exercise in preventing NIDDM in people with impaired glucose tolerance. The Da Qing IGT and Diabetes Study. Diabetes Care 1997;20:537-544.

Peltonen M, Korpi-Hyövälti E, Oksa H, Puolijoki H, Saltevo J, Vanhala M, Saaristo T, Saarikoski L, Sundvall J, Tuomilehto J. Lihavuuden, diabeteksen ja muiden glukoosiaineenvaihdunnan häiriöiden esiintyvyys suomalaisessa aikuisväestössä Dehkon 2D-hanke (D2D). SLL 2006;3:163–170.

[Ramachandran A, Snehalatha C, Mary S, Mukesh B, Bhaskar AD, Vijay V. Indian Diabetes Prevention Programme (IDPP).](http://www.ncbi.nlm.nih.gov/entrez/query.fcgi?cmd=Retrieve&db=pubmed&dopt=Abstract&list_uids=16391903&query_hl=47&itool=pubmed_DocSum) The Indian Diabetes Prevention Programme shows that lifestyle modification and metformin prevent type 2 diabetes in Asian Indian subjects with impaired glucose tolerance (IDPP-1). Diabetologia 2006;49:289-297.

Reaven GM. Banting lecture 1988. Role of insulin resistance in human disease. Diabetes 1988;37:1595-1607.

Sarkkinen E, Korhonen M, Erkkilä A, Ebeling T, Uusitupa M. Effect of apolipoprotein E polymorphism on serum lipid response to the separate modification of dietary fat and dietary cholesterol. Am J Clin Nutr 1998;68:1151-1152.

Schwab US, Ågren JJ, Valve R, Hallikainen MA, Sarkkinen ES, Jauhiainen M, Karvonen MK, Pesonen U, Koulu M, Uusitupa M, Savolainen MJ. The impact of the leucine 7 to proline 7 polymorphism of the neuropeptide Y gene on postprandial lipemia and on the response of serum total and lipoprotein lipids to a reduced fat diet. Eur J Clin Nutr 2002;56:149-156.

Sirtori CR, Crepaldi G, Manzato E, Mancini M, Rivellese A, Paoletti R, Pazzucconi F, Pamparana F, Stragliotto E. One-year treatment with ethyl esters of n-3 fatty acids in patients with hypertriglyceridemia and glucose intolerance. Reduced triglyceridemia, total cholesterol and increased HDL-C without glycemic alterations. Atherosclerosis 1998;137:419-427.

[Tsuda T, Ueno Y, Aoki H, Koda T, Horio F, Takahashi N, Kawada T, Osawa T.](http://www.ncbi.nlm.nih.gov/entrez/query.fcgi?cmd=Retrieve&db=pubmed&dopt=Abstract&list_uids=15003523&query_hl=20&itool=pubmed_docsum) Anthocyanin enhances adipocytokine secretion and adipocyte-specific gene expression in isolated rat adipocytes. Biochem Biophys Res Commun 2004;316:149-157.

[Tsuda T, Ueno Y, Kojo H, Yoshikawa T, Osawa T.](http://www.ncbi.nlm.nih.gov/entrez/query.fcgi?cmd=Retrieve&db=pubmed&dopt=Abstract&list_uids=15863361&query_hl=23&itool=pubmed_docsum) Gene expression profile of isolated rat adipocytes treated with anthocyanins. Biochim Biophys Acta 2005;1733:137-147.

[Tsuda T, Ueno Y, Yoshikawa T, Kojo H, Osawa T.](http://www.ncbi.nlm.nih.gov/entrez/query.fcgi?cmd=Retrieve&db=pubmed&dopt=Abstract&list_uids=16483547&query_hl=24&itool=pubmed_docsum) Microarray profiling of gene expression in human adipocytes in response to anthocyanins. Biochem Pharmacol 2006;71:1184-1197.

[Tuomilehto J, Lindström J, Eriksson JG, Valle TT, Hämäläinen H, Ilanne-Parikka P, Keinänen-Kiukaanniemi S, Laakso M, Louheranta A, Rastas M, Salminen V, Uusitupa M. Finnish Diabetes Prevention Study Group.](http://www.ncbi.nlm.nih.gov/entrez/query.fcgi?cmd=Retrieve&db=pubmed&dopt=Abstract&list_uids=11333990&query_hl=38&itool=pubmed_DocSum) Prevention of type 2 diabetes mellitus by changes in lifestyle among subjects with impaired glucose tolerance. N Engl J Med 2001;344:1343-1350.

[Uusitupa M.](http://www.ncbi.nlm.nih.gov/entrez/query.fcgi?cmd=Retrieve&db=pubmed&dopt=Abstract&list_uids=15955472&query_hl=29&itool=pubmed_docsum) Gene-diet interaction in relation to the prevention of obesity and type 2 diabetes: evidence from the Finnish Diabetes Prevention Study. Nutr Metab Cardiovasc Dis 2005;15:225-233.

[Uusitupa M, Lindi V, Louheranta A, Salopuro T, Lindström J, Tuomilehto J; Finnish Diabetes Prevention Study Group.](http://www.ncbi.nlm.nih.gov/entrez/query.fcgi?cmd=Retrieve&db=pubmed&dopt=Abstract&list_uids=14514637&query_hl=30&itool=pubmed_docsum) Long-term improvement in insulin sensitivity by changing lifestyles of people with impaired glucose tolerance: 4-year results from the Finnish Diabetes Prevention Study. [Diabetes](javascript:AL_get(this, 'jour', 'Diabetes.');) 2003;52:2532-2538.

[Uusitupa MI, Niskanen LK, Siitonen O, Voutilainen E, Pyörälä K.](http://www.ncbi.nlm.nih.gov/entrez/query.fcgi?cmd=Retrieve&db=pubmed&dopt=Abstract&list_uids=8270133&query_hl=12&itool=pubmed_docsum) Ten-year cardiovascular mortality in relation to risk factors and abnormalities in lipoprotein composition in type 2 (non-insulin-dependent) diabetic and non-diabetic subjects. Diabetologia. 1993;36:1175-1184.

Vessby B, Uusitupa M, Hermansen K, Riccardi G, Rivellese AA, Tapsell LC, Nalsen C, Berglund L, Louheranta A, Rasmussen BM, Calvert GD, Maffetone A, Pedersen E, Gustafsson IB, Storlien LH; KANWU Study. Substituting dietary saturated for monounsaturated fat impairs insulin sensitivity in healthy men and women: The KANWU Study. Diabetologia. 2001;44:312-319.

Woodman RJ, Mori TA, Burke V, Puddey IB, Watts GF, Beilin LJ. Effects of purified eicosapentaenoic and docosahexaenoic acids on glycemic control, blood pressure, and serum lipids in type 2 diabetic patients with treated hypertension. Am. J. Clin. Nutr. 2002;76:1007-1015.

Yudkin JS. Adipose tissue, insulin action and vascular disease: inflammatory signals. Int J Obes Relat Metab Disord 2003;27 (Suppl 3):S25-S28.
